# Origin and divergence of Afro-Indian Picrodendraceae: linking pollen morphology, dispersal modes, fossil records, molecular dating and paleogeography

**DOI:** 10.1080/00173134.2019.1594357

**Published:** 2019-06-10

**Authors:** Friðgeir Grímsson, Shirley A. Graham, Mario Coiro, Bonnie F. Jacobs, Alexandros Xafis, Frank H. Neumann, Louis Scott, Jakub Sakala, Ellen D. Currano, Reinhard Zetter

**Affiliations:** 1 Department of Botany and Biodiversity Research, University of Vienna, Vienna, Austria; 2 Missouri Botanical Garden, St. Louis, MO, USA; 3 Department of Systematic and Evolutionary Botany, University of Zurich, Zurich, Switzerland; 4 Roy M. Huffington Department of Earth Sciences, Southern Methodist University, Dallas, TX, USA; 5 Department of Paleontology, University of Vienna, Vienna, Austria; 6 Evolutionary Studies Institute, University of the Witwatersrand, Johannesburg, South Africa; 7 Department of Plant Sciences, University of the Free State, Bloemfontein, South Africa; 8 Institute of Geology and Palaeontology, Faculty of Science, Charles University, Prague, Czech Republic; 9 Departments of Botany and Geology & Geophysics, University of Wyoming, Laramie, WY, USA

**Keywords:** *Androstachys*, autochory, *Aristogeitonia*, *Hyaenanche*, myrmecochory, *Oldfieldia*, *Piranhea*, *Stachyandra*, *Tetracoccus*, *Voatamalo*

## Abstract

The pantropical Picrodendraceae produce mostly spheroidal to slightly oblate, echinate pollen grains equipped with narrow circular to elliptic pori that can be hard to identify to family level in both extant and fossil material using light microscopy only. Fossil pollen of the family have been described from the Paleogene of America, Antarctica, Australia, New Zealand, and Europe, but until now none have been reported from Afro-India. Extant pollen described here include representatives from all recent Picrodendraceae genera naturally occurring in Africa and/or Madagascar and south India and selected closely related tropical American taxa. Our analyses, using combined light microscopy and scanning electron microscopy, show that pollen of the Afro-Indian genera encompass three morphological types: Type 1, comprising only *Hyaenanche*; Type 2, including *Aristogeitonia*, *Mischodon, Oldfieldia* and *Voatamalo*; Type 3, comprising the remaining two genera, *Androstachys* and *Stachyandra*. Based on the pollen morphology presented here it is evident that some previous light microscopic accounts of spherical and echinate fossil pollen affiliated with Arecaceae, Asteraceae, Malvaceae, and Myristicaceae from the African continent could belong to Picrodendraceae. The pollen morphology of Picrodendraceae, fossil pollen records, a dated intra-familial phylogeny, seed dispersal modes, and the regional Late Cretaceous to early Cenozoic paleogeography, together suggest the family originated in the Americas and dispersed from southern America across Antarctica and into Australasia. A second dispersal route is believed to have occurred from the Americas into continental Africa via the North Atlantic Land Bridge and Europe.

Picrodendraceae are a small pantropical woody family of 25 genera and 96 species in the order Malpighiales (WCSP 2018; see Supporting Information). Prior to changes in the classification of orders and families of flowering plants as a result of increased morphological, anatomical, and especially molecular phylogenetic information, the genera constituted the three tribes of subfamily Oldfieldioideae in the Euphorbiaceae *sensu lato* (Webster 1994a, 1994b). The distribution of Picrodendraceae is disjunct across the subtropical and tropical Southern Hemisphere, with some limited representation in cooler temperate zones. Representatives of the family occur in the Americas (five genera), Africa (six genera; Table I), south India and Sri Lanka (one genus; Table I) and in Australasia (13 genera; see Supporting Information). The greatest diversity at both the genus and species levels occurs in Australia with ten genera (seven endemic) and approximately 40 spp (WCSP 2018). The largest genus is *Austrobuxus* with 22 spp, of which 15 are endemic to New Caledonia (WCSP 2018; see Supporting Information). The extensive vegetative diversity of Picrodendraceae is reflected in the widely different habitats and environments the family occupies (noted from herbarium sheets examined for this study): a range of altitudes from near sea level to over 1800 m; in flooded forests in the Amazon; deserts in the south-western United States and northern Mexico; dry deciduous forests, humid evergreen forests, woodlands, in sand and on rocky hills in Africa, Madagascar, and south India; dry fynbos and savanna in South Africa (Dyer 1975); drier rainforests and woodlands on sandy or serpentine soils in Australia; and rain forests in New Caledonia.

**Table I.** Afro-Indian Picrodendraceae and their distribution.

Genus	Species number	Taxon	Occurence	Geographic region	Pollen samples used for this study
*Androstachys*	1	*A*. *johnsonii* Prain	Mozambique to South Africa, Madagascar	Africa	van der Schyff, 978 (PRE)
*Aristogeitonia*	7	*A. gabonica* Breteler	Gabon	Africa	Sosef, 1794 (MO)
		*A. limoniifolia* Prain	Angola	Africa	
		*A. lophirifolia* Radcl.-Sm.	North and west Madagascar	Madagascar	Capuron, 23.156-SF (MO)
		*A. magnistipula* Radcl.-Sm.	Tanzania	Africa	
		*A. monophylla* Airy Shaw	Southeast Kenya, east Tanzania	Africa	Phillipson, 4954 (MO)
		*A. perrieri* (Leandri) Radcl.-Sm.	North and west Madagascar	Madagascar	Capuron, 18.462-SF (MO)
		*A. uapacifolia* Radcl.-Sm.	Madagascar (Toliara Province)	Madagascar	
*Hyaenanche*	1	*H. globosa* (Gaert.) Lamb. & Vahl	Cape Province in South Africa	Africa	Hall 3912 (PRE); unknown s.n. (WAG)
*Mischodon*	1	*M. zeylanicus* Thwaites	Sri Lanka, South India, Andaman Is.	India	unknown s.n. (WAG)
*Oldfieldia*	4	*O. africana* Benth. & Hook.f.	West and west/central tropical Africa	Africa	Small 621 (K); Leeuwenberg 3780 (WAG)
		*O. dactylophylla* (Welw. ex Oliv.) J.Léonard	Tanzania to south tropical Africa	Africa	Meuangulango 1279 (MO)
		*O. macrocarpa* J.Léonard	Central and southeast DR Congo	Africa	
		*O. somalensis* (Chiov.) Milne-Redh.	Somalia to Mozambique	Africa	Bally 6880 (EA)
*Stachyandra*	4	*S. imberbis* (Air Shaw) Radcl.-Sm.	Northwest Madagascar	Madagascar	
		*S. merana* (Airy Shaw) J.-F.Leroy ex Radcl.-Sm.	Northwest Madagascar	Madagascar	Capuron, 23.335-SF (MO)
		*S. rufibarbis* (Airy Shaw) Radcl.-Sm.	Northeast Madagascar	Madagascar	
		*S. viticifolia* (Airy Shaw) Radcl.-Sm.	Northwest Madagascar	Madagascar	
*Voatamalo*	2	*V. capuronii* Bosser	Northeast Madagascar	Madagascar	
		*V. eugenioides* Capuron ex Bosser	East Madagascar	Madagascar	Capuron, 22.327-SF (MO)

Note: Data extracted from WCSP (2018).

Picrodendraceae includes monoecious or dioecious subshrubs, shrubs, and small to large trees with simple or palmately-compound leaves; tiny, apetalous, mostly unisexual flowers; anthers of male flowers few or to as many as 55; ovaries 2–7-locular with biovulate locules; fruit capsular and dehiscent or drupaceous; seeds one or two per locule; and copious endosperm. The floral structure indicates a close relationship to the Phyllanthaceae (formerly fam. Euphorbiaceae s.l., subfam. Phyllanthoideae), which is supported in molecular phylogenetic analyses of the Euphorbiaceae (Wurdack et al. 2004; Wurdack & Davis 2009) and Malpighiales (Soltis et al. 2011; Xi et al. 2012). Morphologically, Picrodendraceae and Phyllanthaceae are united by unspecialised monopodial branching patterns, a lack of laticifers, and biovulate locules in the capsular fruit (Webster 1994a, 1994b; Merino Sutter et al. 2006). In an extensive phylogeny of the order Malpighiales, sequences of 82 plastid genes produced a fully supported tree in which Picrodendraceae and Phyllanthaceae were confirmed as sister families (Xi et al. 2012). The South American genus *Podocalyx* was retrieved as sister to the other Picrodendraceae, with *Tetracoccus* + *Androstachys*, sister to a clade of *Petalostigma, Dissiliaria*, and *Micrantheum* + *Austrobuxus*.

The unique morphology of Picrodendraceae pollen (± spherical, echinate, stephanoporate) and its systematic value was first pointed out by Erdtman (1952) and later by Punt (1962) and Köhler (1965). The pollen morphology of Afro-Indian Picrodendraceae has been studied to some extent (Table II). Among 20 species, eight have been studied using transmission electron microscopy (TEM) (Hayden et al. 1984; Levin & Simpson 1994; Simpson & Levin 1994), six have been studied using scanning electron microscopy (SEM) (Hayden et al. 1984; Levin & Simpson 1994; Lobreau-Callen & Cervera 1994; Simpson & Levin 1994), and four have been illustrated using light microscopy (LM) micrographs (Köhler 1965). These studies documented some variation in the pollen morphology among genera or at the species level of Afro-Indian Picrodendraceae that suggested fossil Picrodendraceae pollen grains could be associated with specific intrafamilial lineages.

**Table II.** Previously illustrated pollen of Afro-Indian Picrodendraceae species.

Taxon (Type)	Figured in	Micrograph Nr.	Micrograph type
*Androstachys johnsonii*	Simpson & Levin 1994 (Levin & Simpson 1994)	Fig. 35 (Fig. 7)	SEM, overview
	Simpson & Levin 1994 (Levin & Simpson 1994)	Fig. 36 (Fig. 8)	SEM, close-up
	Simpson & Levin 1994 (Levin & Simpson 1994)	Fig. 45 (Fig. 22)	TEM, non-apertural wall
	Simpson & Levin 1994	Fig. 46	TEM, apertural region
	Köhler 1965	Plate 9, fig. 9	LM, overview
	Punt 1962	Plate VII, fig. 1	LM (drawing)
*Aristogeitonia limoniifolia*	Punt 1962	Plate VI, fig. 5	LM (drawing)
*Aristogeitonia monophylla*	Simpson & Levin 1994	Fig. 52	TEM, non-apertural wall
	Simpson & Levin 1994	Fig. 53	TEM, apertural region
*Aristogeitonia perrieri*	Lobreau-Callen & Cervera 1994	Plate 5, fig. G	SEM, overview
	Lobreau-Callen & Cervera 1994	Plate 5, fig. H	SEM, close-up
	Lobreau-Callen & Cervera 1994	Plate 5, fig. I	SEM, break in wall
*Hyaenanche globosa*	Simpson & Levin 1994	Fig. 54	TEM, non-apertural wall
	Simpson & Levin 1994	Fig. 55	TEM, apertural region
	Punt 1987	Fig. 7	SEM, overview
	Köhler 1965	Plate 8, fig. 18	LM, overview
	Punt 1962	Plate 6, fig. 6	LM (drawing)
*Mischodon zeylanicus*	Simpson & Levin 1994	Fig. 51	TEM, apertural region
	Lobreau-Callen & Cervera 1994	Plate 5, fig. A	SEM, overview
	Lobreau-Callen & Cervera 1994	Plate 5, fig. B	SEM, close-up
	Lobreau-Callen & Cervera 1994	Plate 5, fig. C	SEM, break in wall
	Köhler 1965	Plate 8, fig. 17	LM, overview
*Oldfieldia africana*	Simpson & Levin 1994 (Levin & Simpson 1994)	Fig. 44 (Fig. 18)	TEM, non-apertural wall
	Hayden et al. 1984	Fig. 16	SEM, overview
*Oldfieldia dactylophylla*	Köhler 1965	Plate 8, fig. 16	LM, overview
*Stachyandra merana*	Simpson & Levin 1994	Fig. 47	TEM, non-apertural wall
	Lobreau-Callen & Cervera 1994	Plate 5, fig. D	SEM, overview
	Lobreau-Callen & Cervera 1994	Plate 5, fig. E	SEM, close-up
	Lobreau-Callen & Cervera 1994	Plate 5, fig. F	SEM, break in wall
*Stachyandra rufibarbis*	Simpson & Levin 1994	Fig. 48	TEM, non-apertural wall
	Simpson & Levin 1994	Fig. 49	TEM, apertural region
*Voatamalo eugenioides*	Simpson & Levin 1994	Fig. 50	TEM, non-apertural wall

The current disjunct tropical to subtropical distribution of Picrodendraceae implies that it already had a worldwide distribution in early Cenozoic times. This is supported by fossil pollen records from the Paleocene and/or Eocene of Australia (Martin 1974, 1978, 1982), New Zealand (Raine et al. 2011), Europe (Zetter & Hofmann 2008; Hofmann et al. 2011; Zetter et al. 2011) and North America (Tschudy & van Loenen 1970; Tschudy 1973; Graham et al. 2000). Interestingly, there is not a single fossil record of Picrodendraceae from Afro-India.

Here we describe and illustrate pollen from 12 of the 20 (12/20) extant taxa currently accepted in Afro-India (Table III), including: *Androstachys* (1/1), *Aristogeitonia* (4/7), *Hyaenanche* (1/1), *Oldfieldia* (3/4), *Stachyandra* (1/4), *Voatamalo* (1/2) and *Mischodon* (1/1). Pollen of *Tetracoccus* (1/4) and *Piranhea* (2/4), from the Americas, are also portrayed as they represent early branching taxa/lineages of the American-Afro-Indian clade. The extant African pollen grains are compared, grouped by morphological type, and their diagnostic features highlighted. Fossil Picrodendraceae pollen from the Miocene of Africa and their Eocene predecessors from Europe are also described. The morphological traits of extant taxa are here used to associate fossil Picrodendraceae pollen grains from Europe and Africa with extant genera and/or lineages. Further, the pollen morphology is discussed in relation to dispersal methods, the fossil record, paleogeography, and the current molecular phylogenetic framework, to unravel the paleophytogeographic history of this family in Afro-India. A dated phylogeny is presented supporting the hypothesis that Picrodendraceae originated in the Upper Cretaceous of the Americas from where they dispersed across the world during the Cenozoic.

**Table III.** Herbarium material used for this study.

Taxon	Collector	Coll. Nr.	Country	Herbarium
*Androstachys johnsonii* Prain	H.P.v.d. Schyff	978	South Africa	PRE
*Aristogeitonia gabonica* Breteler	M.S.M. Sosef	1794	Gabon	MO
*Aristogeitonia lophirifolia* Radcl.-Sm.	R. Capuron	23.156-SF	Madagascar	MO
*Aristogeitonia monophylla* Airy Shaw	P.B. Phillipson	4954	Tanzania	MO
*Aristogeitonia perrieri* (Leandri) Radcl.-Sm.	R. Capuron	18.462-SF	Madagascar	MO
*Hyaenanche globosa* (Gaert.) Lamb. & Vahl	H. Hall	3912	Namibia	PRE
*Hyaenanche globosa* (Gaert.) Lamb. & Vahl	Unknown	s.n.	South Africa	WAG
*Mischodon zeylanicus* Thwaites	Unknown	s.n.	India	WAG
*Oldfieldia africana* Benth. & Hook.f.	D. Small	621	Sierra Leone	K
*Oldfieldia africana* Benth. & Hook.f.	Leeuwenberg	3780	Ivory Coast	WAG
*Oldfieldia dactylophylla* (Welw. Ex Oliv.) J.Léonard	N.A. Meuangulango	1279	Tanzania	MO
*Oldfieldia somalensis* (Chiov.) Milne-Redh.	P.C. Bally	6880	Tanzania	EA
*Piranhea longepedunculata* Jabl.	Liesner & Gonzalez	5859	Venezuela	WAG
*Piranhea trifoliata* Baill.	Berg et al.	P19790	Brazil	WAG
*Stachyandra merana* (Airy Shaw) J.-F.Leroy ex Radcl.-Sm.	R. Capuron	23.335-SF	Madagascar	MO
*Tetracoccus fasciculatus* (S.Watson) Croizat	Langenberg	s.n.	USA	WAG
*Voatamalo eugenioides* Capuron ex Bosser	R. Capuron	22.327-SF	Madagascar	MO

## Material and methods

### Origin and preparation of samples

Extant flower material (see Table III) from the Missouri Botanical Garden (MO), the Royal Botanic Gardens, Kew (K), the South African National Biodiversity Institute (PRE), the National Museums of Kenya (EA), and Naturalis (WAG) was prepared according to the protocol outlined in Grímsson et al. (2017a, 2018b) and Halbritter et al. (2018).

The fossil Picrodendraceae pollen identified during this study occurred in five different sedimentary rock samples: (1) the Holzer Formation (early Eocene) at Krappfeld, Pemberg Quarry, west of Klein St Paul, Carinthia, Austria, (2) the Borkener coal measures (middle Eocene) of the Stolzenbach underground coal mine, near Kassel, Germany, (3) the Profen Formation (middle Eocene) of the Profen opencast mine, close to Leipzig, Germany, (4) the Mush Valley fossil beds (sample MU07; early Miocene) of the central Ethiopian Plateau, Ethiopia, and (5) the Elandsfontyn Formation (early Miocene), core sample #114755 from Saldanha Bay, South Africa. For details on the geographic positions, geology, palaeoecology, and previously reported fossil plants from these formations and localities see Table IV and references cited therein.

**Table IV.** Information on sample sites.

	Krappfeld MT	Stolzenbach MT	Profen MT	Mush MT	Saldanha MT
Location	Krappfeld area, Carinthia, Austria	Stolzenbach underground coalmine, Kassel, Germany	Profen opencast mine, close to Leipzig, Germany	Mush Valley, Debre Birhan Woreda, Ethiopia	Saldanha Bay drill core, South Africa
Latitude and longitude (*c*.)	46°50′N, 14°31′E	51°0′N, 9°17′E	51°09′N, 12°11′E	9°47′N, 39°39′E	33°01′S, 17°59′E
Lithostratigraphy	Holzer Formation	Borkener coal measures	Profen Formation	Unnamed lacustrine sediments (sample MU07)	Elandsfontyn Formation (core sample #114755)
Epoch^a^	Ypresian	Lutetian	Bartonian	Aquitanian	Aquitanian
Age (Ma)^a^	56.0–47.8^a^	47.8–41.2^a^	41.2–38^a^	22.63–21.73 (absolute dating)	23.03–20.44^a^
Age according to	Litho- and biostratigraphy	Litho- and biostratigraphy	Litho- and biostratigraphy	Chrono- and lithostratigraphy	Litho- and biostratigraphy
For further info on the geological background, stratigraphy (S), paleoenvironment, paleoclimate, and plant fossils (P)	Hofmann & Zetter 2001 (S/P); Zetter & Hofmann 2001 (P); Hofmann et al. 2012, Hofmann et al. 2015a, 2015b (S/P); Hofmann 2018 (P)	Oschkinis & Gregor 1992 (P/S); Gregor 2005 (P); Hottenrott et al. 2010 (P); Gregor & Oschkinis 2013 (P); Manchester et al. 2015 (P); Grímsson et al. 2017b (P); Hofmann 2018 (P), Hofmann & Gregor 2018 (P)	Krutzsch & Lenk 1973 (P/S); Pälchen & Walter 2011 (S); Manchester et al. 2015 (P); Grímsson et al. 2017c (P)	Danehy 2010 (S/P); Pan et al. 2012 (S/P), 2014 (P), Bush et al. 2017(P), Tesfamichael et al. 2017 (S/P)	Roberts et al. 2017 (S/P); Coetzee 1981, 1983 (P); Coetzee & Muller 1984 (P), Coetzee & Praglowski 1984, 1988 (P); Grímsson et al. 2018c (P).

^a^Following ICS (2017)

The sedimentary rock samples were processed and fossil pollen grains extracted according to the method explained in Grímsson et al. (2008). The fossil Picrodendraceae pollen grains were investigated both by LM and SEM using the single grain method as described by Zetter (1989) and Halbritter et al. (2018). SEM stubs with extant and fossil Picrodendraceae pollen produced under this study are stored in the collection of the Department of Paleontology, University of Vienna, Austria.

### Tree inference

Sequences for five mitochondrial loci (*ccmB, cob, nad1, nad6*, mt-*rps3*), three nuclear loci (*18S, EMB2765, PHYC*), and four plastid loci (*atpB, matK, ndhF, rbcL*) from Picrodendraceae were downloaded from NCBI GenBank. Sequences for each locus were aligned using MAFFT v.7.273 (Katoh & Standley 2013) using the L-INS-I algorithm for all loci. Alignments were visually inspected for irregularities and truncated at the end for portions covering data of less than seven genera. The single locus alignments were then concatenated using MESQUITE (Maddison & Maddison 2011). Phylogenetic analyses were run using RaxML v.8.2.10 (Stamatakis 2014), which we used to obtain 1000 (fast) bootstrap replicates, and MrBayes v.3.2.6 (Ronquist et al. 2012), where we conducted two runs with one cold chain and three heated chains for 1 900 000 mcmc generations, with 25% of the generations discarded as burnin. Parameters of the mcmc runs were inspected using Tracer (Rambaut & Drummond 2003) to check for convergence. In both cases, data were partitioned by genome (mitochondrial, plastidial, and nuclear), and an unlinked GTR model with gamma-distributed rate variation was employed. RaxML bootstrap replicates and post-burnin samples from both runs were synthesised using consensus networks (Holland & Moulton 2003) in SplitsTree (Huson & Bryant 2006), using a 20% cutoff.

To obtain a timescale for Picrodendraceae, we conducted a molecular dating analysis with the fossilised birth–death prior (Heath et al. 2014) as implemented in MrBayes v.3.2.6. Fossil pollen taxa compiled for this study were coded as tips, with a uniform prior on their age reflecting uncertainty in the dates. Fossils were constrained in clades with their most probable extant relatives based on informal assessment of their micromorphology. For the molecular data, we employed a relaxed IGR clock, with the same substitution model implemented for the non-clock analysis (see earlier). We set the sampling probability of the extant taxa as 0.26 (based on species count of extant Picrodendraceae), and we set the sample strategy as diversified. We ran two independent runs with four chains (one cold, four heated) for 50 000 000 generations. A consensus tree was generated using all compatible splits.

## Systematic palynology

The pollen terminology follows Punt et al. (2007; LM) and Halbritter et al. (2018; SEM). The classification and author names of extant taxa follow WCSP (2018). Genera and species are arranged in alphabetical order with the pollen of each taxon described individually, followed by descriptions of the fossils at the end. Pollen grains of all the extant American-Afro-Indian Picrodendraceae species studied here are compared in Table V. Three morphological types are recognised, referred to here as PT 1–PT 3. Fossil Picrodendraceae pollen grains are compared in Table VI. For practical reasons all the fossil pollen grains are classified as morphotypes (MT) named after the locality where they were found.

**Table V.** Pollen morphology of extant Picrodendraceae.

	Androstachys johnsonii	Aristogeitonia gabonica	Aristogeitonia lophirifolia	Aristogeitonia monophylla	Aristogeitonia perrieri	Hyaenanche globosa	Mischodon zeylanicus
P/E ratio	Spheroidal to oblate	Spheroidal to slightly oblate	Spheroidal to slightly oblate	Spheroidal to slightly oblate	Spheroidal to slightly oblate	Spheroidal to slightly oblate	Spheroidal to slightly oblate
Outline in polar and equatorial view	**Elliptic** to circular **to slightly angular**	Circular	Circular	Circular	Circular	Circular	Circular
Equatorial diameter including echini (LM)	34–38	27–31	25–29	32–36	27–29	32–39	32–38
Equatorial diameter including echini (SEM)	33–36	26–28	25–28	32–34	25–29	30–38	32–37
Equatorial diameter excluding echini (LM)	N/A	21–25	20–24	25–30	22–25	29–35	27–33
Equatorial diameter excluding echini (SEM)	32–34	21–23	21–23	25–27	21–25	28–34	27–32
Polar axis including echini (LM)	30–33	25–28	25–28	32–35	26–28	30–35	30–34
Polar axis including echini (SEM)	29–32	23–25	23–25	31–33	24–27	29–34	31–34
Polar axis excluding echini (LM)	N/A	20–23	21–23	26–28	22–24	29–33	26–30
Polar axis excluding echini (SEM)	28–31	19–21	19–21	25–27	20–23	26–32	26–28
Aperture type and number	Stephano(5-7)porate	Stephano(5-6)porate	Stephano(6-7)porate	Stephano(7)porate	Stephano(6-7)porate	Stephano(6-7)porate	Stephano(5-7)porate
Aperture position	**Pori** often **at irregular intervals**, one or two pori can be **positioned outside of the equator**	Pori at regular intervals, positioned at the equator	Pori at regular intervals, positioned at the equator	Pori at regular intervals, positioned at the equator	Pori at regular intervals, positioned at the equator	Pori at regular intervals, positioned at the equator	Pori at regular intervals, positioned at the equator
Aperture diameter (SEM)	2.5–4.5	2.5–3.5	2.5–4.0	3.5–5.0	2.5–3.5	3.0–5.5	3.5–4.5
Exine thickness (LM)	1.2–1.3	1.2–1.5	1.1–1.2	1.6–1.7	1.1–1.5	1.4–1.6	1.3–1.8
Pollen wall (SEM)	Tectate	Tectate	Tectate	Tectate	Tectate	Tectate	Tectate
Sculpture (LM)	Echinate	Echinate	Echinate	Echinate	Echinate	Echinate	Echinate
Sculpture (SEM)	**Microechinate; granulate and perforate** in areas between echini	Echinate; nanogemmate to nanorugulate in areas between echini	Echinate; nanogemmate to granulate in areas between echini	Echinate; nanogemmate to nanorugulate in areas between echini	Echinate; nanogemmate to granulate in areas between echini	Echinate; **fossulate, perforate and nanogemmate** in areas between echini	Echinate; nanogemmate to granulate in areas between echini
Number of echini in central polar area (SEM)	**30–45 per 100 µm^2^**	5–11 per 100 µm^2^	9–13 per 100 µm^2^	3–7 per 100 µm^2^	9–12 per 100 µm^2^	**15–30 per 100 µm^2^**	3–7 per 100 µm^2^
Echini height (SEM)	**0.7–1.3**	3.0–4.5	2.0–3.5	3.0–4.5	2.0–3.0	**1.0–2.0**	3.0–4.5
Aperture membrane (SEM)	**Nanoechinate**, nanogemmate and granulate	Nanogemmate to nanorugulate	Nanogemmate to granulate	Nanogemmate to nanorugulate	Nanogemmate to granulate	**Nanogemmate**	Nanogemmate to granulate
Pollen Type	3	2	2	2	2	1	2

Oldfieldia africana	Oldfieldia dactylophylla	Oldfieldia somalensis	Piranhea longepedunculata	Piranhea trifoliata	Stachyandra merana	Tetracoccus fasciculatus	Voatamalo eugenioides
Spheroidal to slightly oblate	Spheroidal to slightly oblate	Spheroidal to slightly oblate	Spheroidal to slightly oblate	Spheroidal to slightly oblate	Spheroidal to oblate	Spheroidal to oblate	Spheroidal to slightly oblate
Circular	Circular	Circular	Circular	Circular	**Elliptic** to circular **to slightly angular**	Circular	Circular
30–34	32–38	30–37	27–30	25–31	32–40	31–35	35–38
26–36	28–34	28–35	25–29	24–29	33–38	30–33	28–37
25–29	27–34	25–30	24–27	24–29	N/A	30–33	28–33
22–29	22–27	22–28	22–24	21–26	31–36	28–30	22–31
28–30	31–35	28–32	25–28	24–28	33–35	29–31	32–35
26–28	27–32	29–31	21–24	23–26	33–35	27–28	28–31
23–25	25–28	23–27	22–25	22–25	N/A	27–30	27–32
22–24	21–25	23–25	20–22	21–23	32–34	25–26	23–26
Stephano(6-8)porate	Stephano(7-8)porate	Stephano(6-8)porate	Stephano(6-7)porate	Stephano(6-7)porate	Stephano(4-6)porate	Stephano(6)porate	Stephano(6-7)porate
Pori at regular intervals, positioned at the equator	Pori at regular intervals, positioned at the equator	Pori at regular intervals, positioned at the equator	Pori at regular intervals, positioned at the equator	Pori at regular intervals, positioned at the equator	**Pori** often **at irregular intervals**, one or two pori can be **positioned outside of the equator**	Pori at regular intervals, positioned at the equator	Pori at regular intervals, positioned at the equator
3.0–4.0	3.0–3.5	3.0–4.5	3.6–4.4	3.3–4.2	2.5–4.5	3.0–4.4	2.5–4.0
1.6–1.9	1.5–1.8	1.5–1.7	1.7–2.0	1.7–2.0	1.1–1.3	2.0–2.5	1.3–1.6
Tectate	Tectate	Tectate	Tectate	Tectate	Tectate	Tectate	Tectate
Echinate	Echinate	Echinate	Echinate	Echinate	Echinate	Echinate	Echinate
Echinate; nanogemmate to nanorugulate in areas between echini	Echinate; nanogemmate to nanorugulate to granulate in areas between echini	Echinate; nanogemmate to nanorugulate in areas between echini	Echinate; fossulate, perforate and nanogemmate in areas between echini	Echinate; fossulate, perforate and nanogemmate in areas between echini	**Microechinate; granulate and perforate** in areas between echini	Echinate; nanogemmate to granulate, perforate in areas between echini	Echinate; nanogemmate to nanorugulate in areas between echini
8–12 per 100 µm^2^	6–8 per 100 µm^2^	6–9 per 100 µm^2^	12–16 per 100 µm^2^	13–20 per 100 µm^2^	**30–45 per 100 µm^2^**	20–25 per 100 µm^2^	6–11 per 100 µm^2^
2.0–4.0	3.0–4.0	2.5–3.5	1.6–2.6	1.6–2.7	0.7–1.2	1.1–2.2	2.5–4.0
Nanogemmate to nanorugulate	Nanogemmate to nanorugulate to granulate	Nanogemmate to nanorugulate	Nanogemmate	Nanogemmate	**Nanoechinate**, nanogemmate and granulate	Nanogemmate to granulate	Nanogemmate to nanorugulate
2	2	2	N/A	N/A	3	N/A	2

Note: All measurements include only those from this study and are given in µm. Most diagnostic features appear in bold font. N/A = not applicable.

**Table VI.** Pollen morphology of fossil European and African Picrodendraceae.

	Krappfeld MT	Stolzenbach MT	Profen MT	Mush MT	Saldanha MT
*P/E* ratio	Spheroidal to slightly oblate	Spheroidal to slightly oblate	Spheroidal to slightly oblate	Spheroidal to slightly oblate	Spheroidal to slightly oblate
Outline in polar and equatorial view	Circular	Circular	Circular	Circular	Circular
Equatorial diameter including echini (LM)	23–25	24–32	28–30	40–43	28–38
Equatorial diameter including echini (SEM)	21–23	22–30	26–30	36–39	26–35
Equatorial diameter excluding echini (LM)	20–22	22–30	26–27	32–35	25–35
Equatorial diameter excluding echini (SEM)	16–18	20–28	23–27	31–32	23–32
Polar axis including echini (LM)	19–21	22–30	N/O	34–40	27–30
Polar axis including echini (SEM)	N/O	N/O	N/O	33–35	25–27
Polar axis excluding echini (LM)	15–17	20–28	N/O	26–33	25–28
Polar axis excluding echini (SEM)	N/O	N/O	N/O	29–30	22–24
Aperture type and number	Stephano(7)porate	Stephano(7)porate	Stephano(7)porate	Stephano(5-6)porate	Stephano(7)porate
Aperture position	Pori at regular intervals, positioned at the equator	Pori at regular intervals, positioned at the equator	Pori at regular intervals, positioned at the equator	Pori at regular intervals, positioned at the equator	Pori at regular intervals, positioned at the equator
Aperture diameter (SEM)	3.5–4.0	2.1–2.6	1.0–2.3	2.3–3.1	3.0–4.5
Exine thickness (LM)	1.3–1.5	1.2–1.4	1.2–1.3	2.0–2.2	1.2–1.4
Pollen wall (SEM)	Tectate	Tectate	Tectate	Tectate	Tectate
Sculpture (LM)	Echinate	Echinate	Echinate	Echinate	Echinate
Sculpture (SEM)	Echinate; fossulate, perforate and nanogemmate in areas between echini	Echinate; fossulate, perforate and nanogemmate in areas between echini	Echinate; fossulate, perforate and nanogemmate in areas between echini	Echinate; nanogemmate to naorugulate in areas between echini	Echinate; fossulate, perforate and nanogemmate in areas between echini
Number of echini in central polar area (SEM)	20–25 per 100 µm^2^	19–30 per 100 µm^2^	17–25 per 100 µm^2^	4–8 per 100 µm^2^	15–30 per 100 µm^2^
Echini height (SEM)	2.3–2.6	0.9–2.7	1.7–3.1	4.1–5.2	1.0–2.5
Aperture membrane (SEM)	Nanogemmate	Nanogemmate	Nanogemmate	Nanogemmate to naorugulate	Nanogemmate
Affiliation	Pollen close to *Piranhea*	Pollen close to *Piranhea*	Pollen close to *Piranhea*	Pollen of the *Aristogeitonia*/*Mischodon*/*Oldfieldia*/*Voatamalo* clade	PT 1: aff. *Hyaenanche*

Note: All measurements are given in µm. N/O = not observed.

*Note on the SEM based pollen morphology. *— Fossil European Picrodendraceae pollen grains are characterised by finely striate sculpture on the echini and around the basal area of the echini (e.g. Zetter & Hofmann 2008; Zetter et al. 2011). This feature is hard to see in extant material, but occurs in some of the SEM-studied genera/taxa (see descriptions of *Hyaenanche, Aristogeitonia* and *Oldfieldia*). It is suggestive that all American and Afro-Indian taxa have such striae, but the pollen surface can be covered by a thin sporopollenin layer obscuring this feature. This sporopollenin layer, which appears granulate in SEM, is not easily removed during preparation, but is often eroded during fossilisation revealing the distinctive striate sculpture.

## 

### Pollen descriptions of extant taxa

Family Picrodendraceae

Genus *Androstachys*

Species *Androstachys johnsonii* Prain (van der Schyff, 978 [PRE])

(Figure 1, Table V)

### 

#### Description

Pollen monad, isopolar, polar/equatorial (*P*/*E*) ratio spheroidal to oblate, outline elliptic to circular to slightly angular in polar and equatorial view; equatorial diameter including echini 34–38 µm in LM, 33–36 µm in SEM, equatorial diameter excluding echini 32–34 µm in SEM, polar axis including echini 30–33 µm in LM, 29–32 µm in SEM, polar axis excluding echini 28–31 µm in SEM; stephano(5–7)porate, pori often at irregular intervals, one or two pori sometimes outside of the equator, pori elliptic, 2.5–4.5 µm in diameter (SEM); exine 1.2–1.3 µm thick; pollen wall tectate; sculpture echinate in LM, microechinate in SEM, granulate and perforate in areas between echini (SEM); 30–45 echini per 100 µm^2^ in central polar area, echini at irregular intervals, echini 0.7–1.3 µm in height; aperture membrane nanoechinate, nanogemmate and granulate (SEM).

#### Remarks

The irregular interval and displacement of pori outside of the equator gives the pollen a slightly angular outline in polar and equatorial view. Pollen of this taxon were figured by Köhler (1965, plate 9, figure 9 [LM]) and Simpson and Levin (1994, figures 35, 36 [SEM]). The SEM close-up figured in Simpson and Levin (1994) shows echini that look similar in size and outline to what is observed in the material studied herein, but lacks the resolution needed to identify any sculpture elements in the areas between the echini. Micrographs showing the ultrastructure of *Androstachys* pollen, from both non-apertural and apertural regions, have also been provided by Simpson and Levin (1994, figures 45, 46 [TEM]).

Genus *Aristogeitonia*

Species *Aristogeitonia gabonica* Breteler (Sosef, 1794 [MO])

(Figure 2, Table V)

#### Description

Pollen monad, isopolar, *P*/*E* ratio spheroidal to slightly oblate, outline circular in polar and equatorial view; equatorial diameter including echini 27–31 µm in LM, 26–28 µm in SEM, equatorial diameter excluding echini 21–25 µm in LM, 21–23 µm in SEM, polar axis including echini 25–28 µm in LM, 23–25 µm in SEM, polar axis excluding echini 20–23 µm in LM, 19–21 µm in SEM; stephano(5–6)porate; pori at regular intervals, positioned at the equator, elliptic, 2.5–3.5 µm in diameter (SEM); exine 1.2–1.5 µm thick; pollen wall tectate; sculpture echinate in LM and SEM, nanogemmate to nanorugulate in areas between echini (SEM); 5–11 echini per 100 µm^2^ in central polar area; echini at irregular intervals, 3.0–4.5 µm in height, can be faintly striate especially at base; aperture membrane nanogemmate to nanorugulate (SEM).

Species *Aristogeitonia lophirifolia* Radcl.-Sm. (Capuron, 23.156-SF [MO])

(Figure 3, Table V)

#### Description

Pollen monad, isopolar, *P*/*E* ratio spheroidal, outline circular in polar and equatorial view; equatorial diameter including echini 25–29 µm in LM, 25–28 µm in SEM, equatorial diameter excluding echini 20–24 µm in LM, 21–23 µm in SEM, polar axis including echini 25–28 µm in LM, 23–25 µm in SEM, polar axis excluding echini 21–23 µm in LM, 19–21 µm in SEM; stephano(6–7)porate; pori at regular intervals, positioned at the equator, elliptic, 2.5–4.0 µm in diameter (SEM); exine 1.1–1.2 µm thick; pollen wall tectate; sculpture echinate in LM and SEM, nanogemmate to granulate in areas between echini (SEM); 9–13 echini per 100 µm^2^ in central polar area; echini at irregular intervals, 2.0–3.5 µm in height, can be faintly striate especially at base; aperture membrane nanogemmate to granulate (SEM).

Species *Aristogeitonia monophylla* Airy Shaw (Phillipson, 4954 [MO])

(Figure 4, Table V)

#### Description

Pollen monad, isopolar, *P/E* ratio spheroidal to slightly oblate, outline circular in polar and equatorial view; equatorial diameter including echini 32–36 µm in LM, 32–34 µm in SEM, equatorial diameter excluding echini 25–30 µm in LM, 25–27 µm in SEM, polar axis including echini 32–35 µm in LM, 31–33 µm in SEM, polar axis excluding echini 26–28 µm in LM, 25–27 µm in SEM; stephano(7)porate; pori at regular intervals, positioned at the equator, elliptic, 3.5–5.0 µm in diameter (SEM); exine 1.6–1.7 µm thick; pollen wall tectate; sculpture echinate in LM and SEM, nanogemmate to nanorugulate in areas between echini (SEM); 3–7 echini per 100 µm^2^ in central polar area; echini at irregular intervals, 3.0–4.5 µm in height, can be faintly striate especially at base; aperture membrane nanogemmate to nanorugulate (SEM).

#### Remarks

Micrographs showing the ultrastructure of *Aristogeitonia monophylla* pollen are provided by Simpson and Levin (1994, figures 52, 53 [TEM]).

Species *Aristogeitonia perrieri* (Leandri) Radcl.-Sm. (Capuron, 18.462-SF [MO])

(Figure 5, Table V)

#### Description

Pollen monad, isopolar, *P/E* ratio spheroidal to slightly oblate, outline circular in polar and equatorial view; equatorial diameter including echini 27–29 µm in LM, 25–29 µm in SEM, equatorial diameter excluding echini 22–25 µm in LM, 21–25 µm in SEM, polar axis including echini 26–28 µm in LM, 24–27 µm in SEM, polar axis excluding echini 22–24 µm in LM, 20–23 µm in SEM; stephano(6–7)porate; pori at regular intervals, positioned at the equator, elliptic, 2.5–3.5 µm in diameter (SEM); exine 1.1–1.5 µm thick; pollen wall tectate; sculpture echinate in LM and SEM, nanogemmate to granulate in areas between echini (SEM); 9–12 echini per 100 µm^2^ in central polar area; echini at irregular intervals, 2.0–3.0 µm in height, can be faintly striate especially at base; aperture membrane nanogemmate to granulate (SEM).

#### Remarks

The SEM sculpture of the material presented here corresponds to features detectable in micrographs by Lobreau-Callen and Cervera (1994, plate V, figures G–I) from the same taxon.

Genus *Hyaenanche*

Species *Hyaenanche globosa* (Gaert.) Lamb. et Vahl (Hall, 3912 [PRE]; unknown, s.n. [WAG])

(Figure 6, Table V)

#### Description

Pollen, monad, isopolar, *P/E* ratio spheroidal to slightly oblate, outline circular in polar and equatorial view; equatorial diameter including echini 32–39 µm in LM, 30–38 µm in SEM, equatorial diameter excluding echini 29–35 µm in LM, 28–34 µm in SEM, polar axis including echini 30–35 µm in LM, 29–34 µm in SEM, polar axis excluding echini 29–33 µm in LM, 26–32 µm in SEM; stephano(6–7)porate; pori at regular intervals, positioned at the equator, elliptic, 3.0–5.5 µm in diameter (SEM); exine 1.4–1.6 µm thick; pollen wall tectate; sculpture echinate in LM and SEM, fossulate, perforate and nanogemmate in areas between echini (SEM); 15–30 echini per 100 µm^2^ in central polar area; echini at irregular intervals, 1.0–2.0 µm in height, can be faintly striate especially at base; aperture membrane nanogemmate (SEM).

#### Remarks

Köhler (1965, plate 8, figure 18, as *Toxicodendron globosum*) provides an LM micrograph and Punt (1987, figure 7, as *Hyaenanche capensis*) an SEM overview showing *Hyaenanche globosa* pollen that look similar to the material presented here. Also, micrographs displaying the ultrastructure of this taxon are provided by Simpson and Levin (1994, figures 54, 55 [TEM]).

Genus *Mischodon*

Species *Mischodon zeylanicus* Thwaites (unknown, s.n. [WAG])

(Figure 7, Table V)

#### Description

Pollen monad, isopolar, *P/E* ratio spheroidal to slightly oblate, outline circular in polar and equatorial view; equatorial diameter including echini 32–38 µm in LM, 32–37 µm in SEM, equatorial diameter excluding echini 27–33 µm in LM, 27–32 µm in SEM, polar axis including echini 30–34 µm in LM, 31–34 µm in SEM, polar axis excluding echini 26–30 µm in LM, 26–28 µm in SEM; stephano(5–7)porate; at regular intervals, positioned at the equator, elliptic, 3.5–4.5 µm in diameter (SEM); exine 1.3–1.8 µm thick; pollen wall tectate; sculpture echinate in LM and SEM, nanogemmate to granulate in areas between echini (SEM); 5–10 echini per 100 µm^2^ in central polar area; echini at irregular intervals, 3.0–4.5 µm in height; aperture membrane nanogemmate to granulate (SEM).

#### Remarks

*Mischodon zeylanicus* pollen has been studied previously using LM, SEM and TEM. Köhler (1965, plate 8, figure 17) provides an LM overview that corresponds well to the present observations. SEM micrographs are provided by Lobreau-Callen and Cervera (1994, plate V, figures A–C), showing a single grain in overview, a close-up of aperture regions, as well the wall structure via a break in the pollen wall. The pollen ultrastructure of this taxon is figured in Simpson and Levin (1994, figure 51 [TEM]). The SEM micrographs by Lobreau-Callen and Cervera (1994) illustrate sculpture elements identical to those observed in the pollen material presented here.

Genus *Oldfieldia*

Species *Oldfieldia africana* Benth. et Hook.f. (Small, 621 [K]; Leeuwenberg, 3780 [WAG])

(Figure 8, Table V)

#### Description

Pollen monad, isopolar, *P/E* ratio spheroidal to slightly oblate, outline circular in polar and equatorial view; equatorial diameter including echini 30–34 µm in LM, 26–36 µm in SEM, equatorial diameter excluding echini 25–29 µm in LM, 22–29 µm in SEM, polar axis including echini 28–30 µm in LM, 26–28 µm in SEM, polar axis excluding echini 23–25 µm in LM, 22–24 µm in SEM; stephano(6–8)porate; pori at regular intervals, positioned at the equator, elliptic, 3.0–4.0 µm in diameter (SEM); exine 1.6–1.9 µm thick; pollen wall tectate; sculpture echinate in LM and SEM, nanogemmate to nanorugulate in areas between echini (SEM); 8–12 echini per 100 µm^2^ in central polar area; echini at irregular intervals, 2.0–4.0 µm in height, can be faintly striate especially at base; aperture membrane nanogemmate to nanorugulate (SEM).

#### Remarks

The single SEM overview of an *Oldfieldia africana* pollen grain by Hayden et al. (1984, figure 16) does not allow for detailed comparison with the material presented here. A micrograph showing the ultrastructure of *Oldfieldia africana* pollen is provided by Simpson and Levin (1994, figure 44 [TEM]).

Species *Oldfieldia dactylophylla* (Welw. ex Oliv.) J.Léonard (Meuangulango, 1279 [MO])

(Figure 9, Table V)

#### Description

Pollen monad, isopolar, *P/E* ratio spheroidal to slightly oblate, outline circular in polar and equatorial view; equatorial diameter including echini 32–38 µm in LM, 28–34 µm in SEM, equatorial diameter excluding echini 27–34 µm in LM, 22–27 µm in SEM, polar axis including echini 31–35 µm in LM, 27–32 µm in SEM, polar axis excluding echini 25–28 µm in LM, 21–25 µm in SEM; stephano(7–8)porate; pori at regular intervals, positioned at the equator, elliptic, 3.0–3.5 µm in diameter (SEM); exine 1.5–1.8 µm thick; pollen wall tectate; sculpture echinate in LM and SEM, nanogemmate to nanorugulate to granulate in areas between echini (SEM); 6–8 echini per 100 µm^2^ in central polar area; echini at irregular intervals, 3.0–4.0 µm in height, can be faintly striate especially at base; aperture membrane nanogemmate to nanorugulate to granulate (SEM).

#### Remarks

The LM micrograph of *Oldfieldia dactylophylla* pollen in Köhler (1965, plate 6, figure 16) corresponds to the observations in this study.

Species *Oldfieldia somalensis* (Chiov.) Milne-Redh. (Bally, 6880 [EA])

(Figure 10, Table V)

#### Description

Pollen monad, isopolar, *P*/*E* ratio spheroidal to slightly oblate, outline circular in polar and equatorial view; equatorial diameter including echini 30–37 µm in LM, 28–35 µm in SEM, equatorial diameter excluding echini 25–30 µm in LM, 22–28 µm in SEM, polar axis including echini 28–32 µm in LM, 29–31 µm in SEM, polar axis excluding echini 23–27 µm in LM, 23–25 µm in SEM; stephano(6–8)porate; pori at regular intervals, positioned at the equator, elliptic, 3.0–4.5 µm in diameter (SEM); exine 1.5–1.7 µm thick; pollen wall tectate; sculpture echinate in LM and SEM, nanogemmate to nanorugulate in areas between echini (SEM); 6–9 echini per 100 µm^2^ in central polar area; echini at irregular intervals, 2.5–3.5 µm in height, can be faintly striate especially at base; aperture membrane nanogemmate to nanorugulate (SEM).

Genus *Piranhea* Species *Piranhea longepedunculata* Jabl. (Liesner & Gonzalez, 5859 [WAG])

(Figure 11, Table V)

#### Description

Pollen, monad, isopolar, *P/E* ratio spheroidal to slightly oblate, outline circular in polar and equatorial view; equatorial diameter including echini 27–30 µm in LM, 25–29 µm in SEM, equatorial diameter excluding echini 24–27 µm in LM, 22–24 µm in SEM, polar axis including echini 25–28 µm in LM, 21–24 µm in SEM, polar axis excluding echini 22–25 µm in LM, 20–22 µm in SEM; stephano(6–7)porate; pori at regular intervals, positioned at the equator, elliptic, 3.6–4.4 µm in diameter (SEM); exine 1.7–2.0 µm thick; pollen wall tectate; sculpture echinate in LM and SEM, fossulate, perforate and nanogemmate in areas between echini (SEM); 12–16 echini per 100 µm^2^ in central polar area; echini at irregular intervals, 1.6–2.6 µm in height, can be faintly striate especially at base; aperture membrane nanogemmate (SEM).

#### Remarks

The only SEM overview of an *Piranhea longepedunculata* pollen grain by Hayden et al. (1984, figure 18) corresponds to the material presented here.

Species *Piranhea trifoliata* Baill. (Berg et al., P19790 [WAG])

(Figure 12, Table V)

#### Description

Pollen, monad, isopolar, *P/E* ratio spheroidal to slightly oblate, outline circular in polar and equatorial view; equatorial diameter including echini 25–31 µm in LM, 24–29 µm in SEM, equatorial diameter excluding echini 24–29 µm in LM, 21–26 µm in SEM, polar axis including echini 24–28 µm in LM, 23–26 µm in SEM, polar axis excluding echini 22–25 µm in LM, 21–23 µm in SEM; stephano(6–7)porate; pori at regular intervals, positioned at the equator, elliptic, 3.3–4.2 µm in diameter (SEM); exine 1.7–2.0 µm thick; pollen wall tectate; sculpture echinate in LM and SEM, fossulate, perforate and nanogemmate in areas between echini (SEM); 13–20 echini per 100 µm^2^ in central polar area; echini at irregular intervals, 1.6–2.7 µm in height, can be faintly striate especially at base; aperture membrane nanogemmate (SEM).

#### Remarks

The pollen ultrastructure (non-apertural wall) of this taxon is figured by Simpson and Levin (1994, figure 41 [TEM]).

Genus *Stachyandra*

Species *Stachyandra merana* (Airy Shaw) J.-F.Leroy ex Radcl.-Sm. (Capuron, 23.335-SF [MO])

(Figure 13, Table V)

#### Description

Pollen monad, isopolar, *P/E* ratio spheroidal to oblate, outline elliptic to circular to slightly angular in polar and equatorial view; equatorial diameter including echini 32–40 µm in LM, 33–38 µm in SEM, equatorial diameter excluding echini 31–36 µm in SEM, polar axis including echini 33–35 µm in LM, 33–35 µm in SEM, polar axis excluding echini 32–34 µm in SEM; stephano(4–6)porate; pori often at irregular intervals, one or two can be positioned outside of the equator, pori elliptic, 2.5–4.5 µm in diameter (SEM); exine 1.1–1.3 µm thick; pollen wall tectate; sculpture echinate in LM, microechinate in SEM, granulate and perforate in areas between echini (SEM); 30–45 echini per 100 µm^2^ in central polar area; echini at irregular intervals, 0.7–1.2 µm in height; aperture membrane nanoechinate, nanogemmate and granulate (SEM).

#### Remarks

The SEM overview of a *Stachyandra merana* pollen grain in Lobreau-Callen and Cervera (1994, plate V, figure D, as *Androstachys murana*) looks very similar to those presented here. The close-ups provided (Lobreau-Callen & Cervera 1994, plate V, figures E, F) also suggest that the echini can be much sharper, and the perforation in the areas between the echini can be bigger and more conspicuous than observed in the present material. The ultrastructure of *Stachyandra merana* pollen is figured by Simpson and Levin (1994, figure 47 [TEM]).

Genus *Tetracoccus*

Species *Tetracoccus fasciculatus* (S.Watson) Croizat (Langenberg, s.n. [WAG])

(Figure 14, Table V)

#### Description

Pollen, monad, isopolar, *P/E* ratio spheroidal to slightly oblate, outline circular in polar and equatorial view; equatorial diameter including echini 31–35 µm in LM, 30–33 µm in SEM, equatorial diameter excluding echini 30–33 µm in LM, 28–30 µm in SEM, polar axis including echini 29–31 µm in LM, 27–28 µm in SEM, polar axis excluding echini 27–30 µm in LM, 25–26 µm in SEM; stephano(6)porate; pori at regular intervals, positioned at the equator, elliptic, 3.0–4.4 µm in diameter (SEM); exine 2.0–2.5 µm thick; pollen wall tectate; sculpture echinate in LM and SEM, nanogemmate to granulate, perforate in areas between echini (SEM); 20–25 echini per 100 µm^2^ in central polar area; echini at irregular intervals, 1.1–2.2 µm in height, can be faintly striate especially at base; aperture membrane nanogemmate to granulate (SEM).

Genus *Voatamalo*

Species *Voatamalo eugenioides* Capuron ex Bosser (Capuron, 22.327-SF [MO])

(Figure 15, Table V)

#### Description

Pollen monad, isopolar, *P/E* ratio spheroidal to slightly oblate, outline circular in polar and equatorial view; equatorial diameter including echini 35–38 µm in LM, 28–37 µm in SEM, equatorial diameter excluding echini 28–33 µm in LM, 22–31 µm in SEM, polar axis including echini 32–35 µm in LM, 28–31 µm in SEM, polar axis excluding echini 27–32 µm in LM, 23–26 µm in SEM; stephano(6–7)porate; pori at regular intervals, positioned at the equator, elliptic, 2.5–4.0 µm in diameter (SEM); exine 1.3–1.6 µm thick; pollen wall tectate; sculpture echinate in LM and SEM, nanogemmate to nanorugulate in areas between echini (SEM); 6–11 echini per 100 µm^2^ in central polar area; echini at irregular intervals, 2.5–4.0 µm in height; aperture membrane nanogemmate to nanorugulate (SEM).

#### Remarks

The ultrastructure of *Voatamalo eugenioides* pollen is illustrated by Simpson and Levin (1994, figure 50 [TEM]).

## 

### Pollen descriptions of fossil taxa

Krappfeld MT, pollen close to *Piranhea*

(Figure 16, Table VI)

### 

#### Description

Pollen, monad, isopolar, *P/E* ratio spheroidal to slightly oblate, outline circular in polar and equatorial view; equatorial diameter including echini 23–25 µm in LM, 21–23 µm in SEM, equatorial diameter excluding echini 22–23 µm in LM, 20–22 µm in SEM, polar axis including echini 19–21 µm in LM, not observed in SEM, polar axis excluding echini 15–17 µm in LM, not observed in SEM; stephano(7)porate; pori at regular intervals, positioned at the equator, elliptic, 3.5–4.0 µm in diameter (SEM); exine 1.3–1.5 µm thick; pollen wall tectate; sculpture echinate in LM and SEM, fossulate, perforate and nanogemmate in areas between echini (SEM); 20–25 echini per 100 µm^2^ in central polar area; echini at irregular intervals, 2.3–2.6 µm in height, striate at base; aperture membrane nanogemmate (SEM).

#### Locality

Krappfeld, Pemberg Quarry, west of Klein St Paul, Carinthia, Austria, early Eocene (Table IV).

#### Remarks

This is an extremely rare element in the Krappfeld palynoflora. The measurements presented earlier are based on a single specimen (versus 20 in extant material) and most likely do not convey the complete natural size ranges of this MT. The Krappfeld MT is not identical to pollen from any extant African Picrodendraceae, but shares most features with the African *Hyaenanche* and especially the American *Piranhea*. The Krappfeld MT pollen in size is closer to that of *Piranhea* than to the larger *Hyaenanche* pollen, and it also has numerous prominent nanogemmae that seem to encircle perforations as in *Piranhea*. In fact, the main difference discriminating the Krappfeld MT pollen grain from those of *Piranhea* is the shape and outline of echini and especially their prominently striate surface. The pollen morphology suggests that the Krappfeld MT represents an extinct early diverging taxon of the American-Afro-Indian clade, positioned close to the *Piranhea* lineage, and is ancestral form leading to the Afro-Indian clade (including *Hyaenanche*). The fossil Picrodendraceae pollen from the Eocene of Austria (Krappfeld MT) and Germany (Stolzenbach MT and Profen MT) are very similar. They clearly represent a closely related stock and might even originate from the same biological taxon.

Stolzenbach MT, pollen close to *Piranhea*

(Figures 16, 17, Table VI)

#### Description

Pollen, monad, isopolar, *P/E* ratio spheroidal to slightly oblate, outline circular in polar and equatorial view; equatorial diameter including echini 24–32 µm in LM, 22–30 µm in SEM, equatorial diameter excluding echini 22–30 µm in LM, 20–28 µm in SEM, polar axis including echini 22–30 µm in LM, not observed in SEM, polar axis excluding echini 20–28 µm in LM, not observed in SEM; stephano(7)porate; pori at regular intervals, positioned at the equator, elliptic, 2.1–2.6 µm in diameter (SEM); exine 1.2–1.4 µm thick; pollen wall tectate; sculpture echinate in LM and SEM, fossulate, perforate and nanogemmate in areas between echini (SEM); 19–30 echini per 100 µm^2^ in central polar area; echini at irregular intervals, 0.9–2.7 µm in height, striate at base; aperture membrane nanogemmate (SEM).

#### Locality

Stolzenbach underground coalmine, Kassel, Germany, middle Eocene (Table IV).

#### Remarks

This is a rare element in the Stolzenbach palynoflora. The measurements presented earlier are based on four specimens (versus 20 in extant material) and most likely do not convey the complete natural size ranges of this MT. For further notes see ‘Remarks’ of the Krappfeld MT.

Profen MT, pollen close to *Piranhea*

(Figure 18, Table VI)

#### Description

Pollen, monad, isopolar, *P/E* ratio spheroidal to slightly oblate, outline circular in polar and equatorial view; equatorial diameter including echini 28–30 µm in LM, 26–30 µm in SEM, equatorial diameter excluding echini 26–27 µm in LM, 23–27 µm in SEM, polar axis including echini not observed in LM or SEM, polar axis excluding echini not observed in LM or SEM; stephano(7)porate; pori at regular intervals, positioned at the equator, elliptic, 1.0–1.5 µm in diameter (SEM); exine 1.2–1.3 µm thick; pollen wall tectate; sculpture echinate in LM and SEM, fossulate, perforate and nanogemmate in areas between echini (SEM); 17–25 echini per 100 µm^2^ in central polar area; echini at irregular intervals, 1.7–3.1 µm in height, striate at base; aperture membrane nanogemmate (SEM).

#### Locality

Profen, Leipzig, central Germany, middle Eocene (Table IV).

#### Remarks

This is an extremely rare element in the Profen palynoflora. The measurements presented earlier are based on a single specimen (versus 20 in extant material) and most likely do not convey the complete natural size ranges of this MT. For further notes see ‘Remarks’ of the Krappfeld MT.

Mush MT, pollen of the *Aristogeitonia*/*Mischodon*/*Oldfieldia*/*Voatamalo* clade

(Figures 19, 20, Table VI)

#### Description

Pollen monad, isopolar, *P/E* ratio spheroidal to slightly oblate, outline circular in polar and equatorial view; equatorial diameter including echini 40–43 µm in LM, 36–39 µm in SEM, equatorial diameter excluding echini 32–35 µm in LM, 31–32 µm in SEM, polar axis including echini 34–40 µm in LM, 33–35 µm in SEM, polar axis excluding echini 26–33 µm in LM, 29–30 µm in SEM; stephano(5–7)porate; pori at regular intervals, positioned at the equator, elliptic, 2.3–3.1 µm in diameter (SEM); exine 2.0–2.2 µm thick; pollen wall tectate; sculpture echinate in LM and SEM, nanogemmate to nanorugulate in areas between echini (SEM); 4–8 echini per 100 µm^2^ in central polar area; echini at irregular intervals, 4.1–5.2 µm in height, can be faintly striate especially at base; aperture membrane nanogemmate to nanorugulate (SEM).

#### Locality

Mush Valley, Debre Birhan Woreda, Ethiopia, early Miocene (Table IV).

#### Remarks

This is not a rare element in the Mush palynoflora and can be found in various stages, in both perfect preservation or compressed and/or broken. The combined features observed in LM and SEM (± spherical, stephanoporate, elliptic pori, echinate sculpture) clearly place these fossil pollen grains in Picrodendraceae. The fossil Mush MT differs considerably from that of *Hyaenanche globosa* (PT 1). The fossil pollen grain is larger, it has much fewer echini per 100 µm^2^ in the central polar area (4–8 versus 15–30) and the echini are also higher (4.1–5.2 versus 1.0–2.0 µm). The sculpture between the echini is nanogemmate to nanorugulate in the Mush MT, but fossulate, perforate, and nanogemmate in *Hyaenanche*. The Mush MT also differs in outline from pollen of *Androstachys* (PT 3) and *Stachyandra* (PT 3) (circular versus elliptic to slightly angular) and in aperture position (regular intervals and at the equator versus irregular intervals and displaced). The echini are also much higher in the Mush MT (4.1–5.2 versus 0.7–1.3 µm) and of different shape and wider apart (4–8 per 100 µm^2^ versus 35–45 per 100 µm^2^) than in both *Androstachys* and *Stachyandra*. Also, the SEM sculpture is nanogemmate to nanorugulate in the Mush MT, but clearly granulate and perforate in *Androstachys* and *Stachyandra* (Tables V, VI). The Mush MT shares many features with extant pollen of PT 2 (*Aristogeitonia, Mischodon, Oldfieldia* and *Voatamalo*). The pollen body of the Mush MT is very large, between 32 and 35 µm in diameter (excluding echini); similar sized pollen is observed in extant *Aristogeitonia monophylla, Mischodon, Oldfieldia*, and *Voatamalo eugenioides* (Table V). All these extant genera also display few (less than 15 per 100 µm^2^) widely spaced echini in the central polar area, that are relatively high (up to 4.5 µm), a feature also characteristic for the Mush MT. The SEM sculpture observed between the echini in the Mush MT is also very similar to that of PT 2 pollen. There is one prominent difference separating the fossil Mush MT from the extant PT 2 pollen and that is the massive thickness of the solid nexine in the Mush MT (see SEM break in Figure 20D, H, J). The pollen morphology suggests that the Mush MT represents a diverging taxon of the Afro-Indian clade, demonstrating an ancestral or extinct form of the *Aristogeitonia-Mischodon-Oldfieldia-Voatamalo* clade.

Saldanha MT, aff. *Hyaenanche*

(Figures 21, 22, Table VI)

#### Description

Pollen, monad, isopolar, *P/E* ratio spheroidal to slightly oblate, outline circular to elliptic in polar and equatorial view; equatorial diameter including echini 28–38 µm in LM, 26–35 µm in SEM, equatorial diameter excluding echini 25–35 µm in LM, 23–32 µm in SEM, polar axis including echini 27–30 µm in LM, 25–27 µm in SEM, polar axis excluding echini 25–28 µm in LM, 22–24 µm in SEM; stephano(6–7)porate; pori at regular intervals, positioned at the equator, elliptic, 3.0–4.5 µm in diameter (SEM); exine 1.2–1.4 µm thick; pollen wall tectate; sculpture echinate in LM and SEM, fossulate, perforate and nanogemmate in areas between echini (SEM); 15–30 echini per 100 µm^2^ in central polar area; echini at irregular intervals, 1.0–2.5 µm in height; aperture membrane nanogemmate (SEM).

#### Locality

Saldanha Bay drill core, South Africa, early Miocene (Table IV).

#### Remarks

Most of the fossil pollen grains are infolded or compressed (flattened). The range of the polar axis is based on only two measurements (versus 20 in extant material) and most likely does not convey the complete natural length between the poles in this MT.

Based on the pollen morphology of extant Afro-Indian Picrodendraceae genera it is clear that the pollen should be assigned to genus *Hyaenanche* (PT 1). The fossil Saldanha MT pollen grains differ in outline from those of *Androstachys* and *Stachyandra* (PT 3; elliptic to slightly angular versus circular) and in aperture position (irregular intervals and displaced versus regular intervals and at the equator; Tables V, VI). The echini are also shorter (0.7–1.3 versus 1.0–2.5 µm), of different shape and more densely packed (35–45 per 100 µm^2^ versus 15–30 per 100 µm^2^) in both *Androstachys* and *Stachyandra* than in the fossil pollen. The SEM sculpture in areas between echini is granulate and perforate in *Androstachys* and *Stachyandra*, but clearly fossulate, perforate and nanogemmate in the fossil pollen (Tables V, VI). The fossil Saldanha MT pollen grains differ also from those of *Aristogeitonia, Mischodon, Oldfieldia* and *Voatamalo* (PT 2). The four genera produce pollen grains that have fewer than 15 echini per 100 µm^2^ in the central polar area, whereas the fossil pollen grains have 15–30 echini per 100 µm^2^ at the central pole. Most of these extant taxa also produce pollen grains equipped with higher echini, 2.0–4.5 µm in height; the echini of the fossil pollen are usually between 1.0 and 2.0 µm and sometimes reach 2.5 µm. The SEM sculpture in areas between the echini is nanogemmate to nanorugulate to granulate in *Aristogeitonia, Mischodon, Oldfieldia* and *Voatamalo*, but fossulate, perforate and nanogemmate in extant *Hyaenanche* (Table V). The fossil Saldanha MT pollen grains are extremely similar to those from the extant species, *Hyaenanche globosa*. The outline and size of the pollen are similar, the position, number, size and outline of the pori are also the same, and they show the same sculpture in SEM. The echini are of similar size, shape and number in the central polar area, the sculpture in areas between echini compares closely, and the preserved parts of the aperture membrane observed in the fossil pollen suggest that it is the same as in the extant species (Tables V, VI).

## Discussion

### Taxonomic value of Afro-Indian Picrodendraceae pollen

Extant Afro-Indian Picrodendraceae pollen can be divided into three morphological types, PT 1–PT 3. The pollen of *Hyaenanche* (PT 1) is clearly unique among the Afro-Indian taxa. The sculpture is echinate, but fossulate, perforate and nanogemmate in areas between echini, and there are 15–30 echini per 100 µm^2^ in the central polar area (Figure 6, Table V). The echini are of intermediate size (1.0–2.0 µm high) and the aperture membranes are nanogemmate.

Many of the Afro-Indian genera produce PT 2. This includes, *Aristogeitonia* (Figures 2–5), *Mischodon* (Figure 7), *Oldfieldia* (Figures 8–10), and *Voatamalo* (Figure 15). PT 2 is mostly spheroidal (*P/E* ratio) and circular in outline (polar and equatorial). The pori are placed at regular intervals around the equator. The sculpture is echinate, with tall (2.0–4.5 µm) but few (3–13 per 100 µm^2^) echini, and nanogemmate to granulate in areas between echini and on the aperture membrane. There are some subtle differences observed among species in the size of the PT 2 pollen grains and their sculpture elements, but there is a complete overlap among the genera (Table V). Taxa from the same geographic region (e.g. *Voatamalo* versus *Aristogeitonia* on Madagascar; *Oldfieldia* versus *Aristogeitonia* in eastern Africa; Figure 23) are impossible to distinguish on the basis of their dispersed pollen.

**Figure 1.**
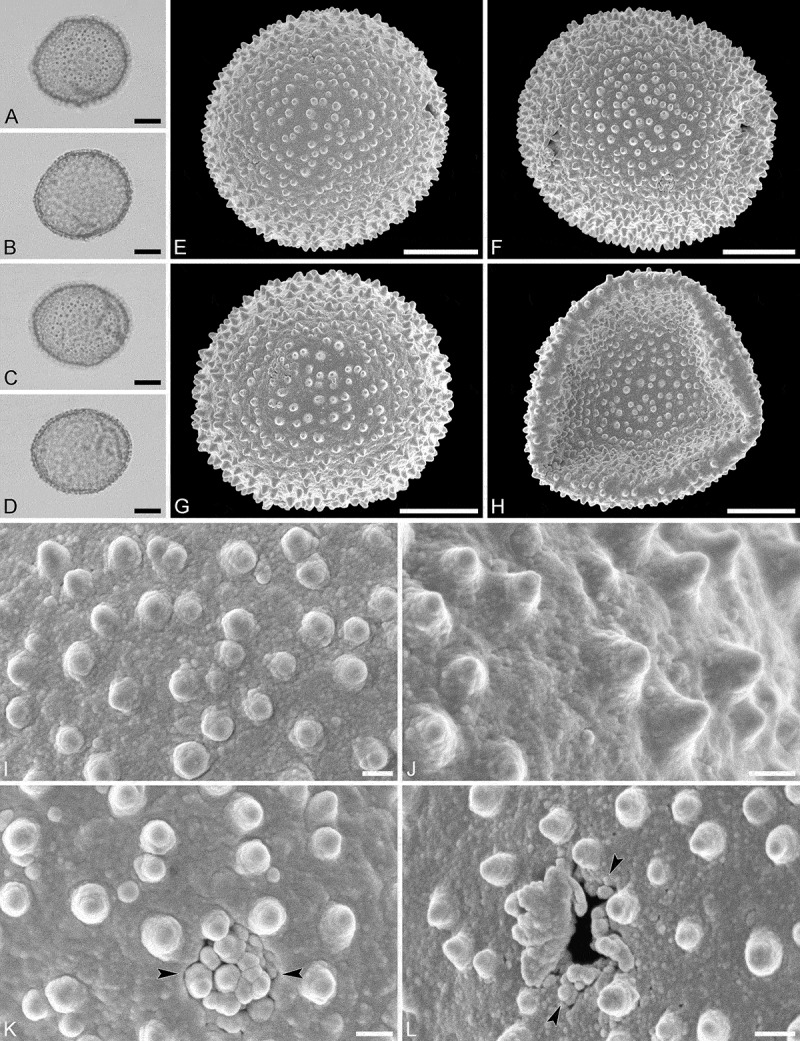
Light microscopy (**A–D**) and scanning electron microscopy (**E–L**) micrographs of *Androstachys johnsonii* (from South Africa, coll. van der Schyff, 978 [PRE]). **A.** Polar view, high focus. **B.** Polar view, optical cross-section. **C.** Equatorial view, high focus. **D.** Equatorial view, optical cross-section. **E.** Oblique polar view. **F.** Oblique equatorial view. **G.** Polar view, note displaced porus. **H.** Infolded grain. **I.** Close-up of interapertural area. **J.** Close-up of interapertural area. **K.** Close-up of aperture, showing membrane (*arrows*). **L.** Close-up of aperture, showing membrane (*arrows*). Scale bars – 10 µm (A–H), 1 µm (I–L).

**Figure 2.**
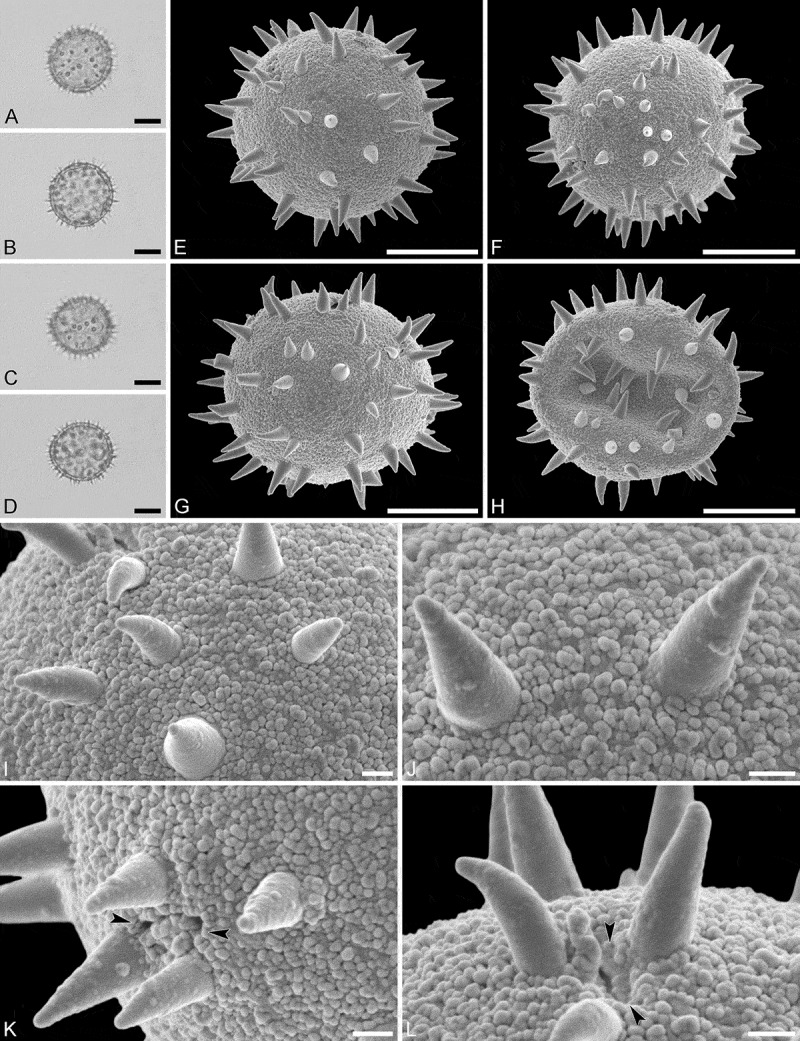
Light microscopy (**A–D**) and scanning electron microscopy (**E–L**) micrographs of *Aristogeitonia gabonica* (from Gabon, coll. Sosef, 1794 [MO]). **A.** Polar view, high focus. **B.** Polar view, optical cross-section. **C.** Equatorial view, high focus. **D.** Equatorial view, optical cross-section. **E.** Polar view. **F.** Polar view. **G.** Polar view. **H.** Infolded grain. **I.** Close-up of interapertural area. **J.** Close-up of interapertural area. **K.** Close-up of aperture, showing membrane (*arrows*). **L.** Close-up of aperture, showing membrane (*arrows*). Scale bars – 10 µm (A–H), 1 µm (I–L).

**Figure 3.**
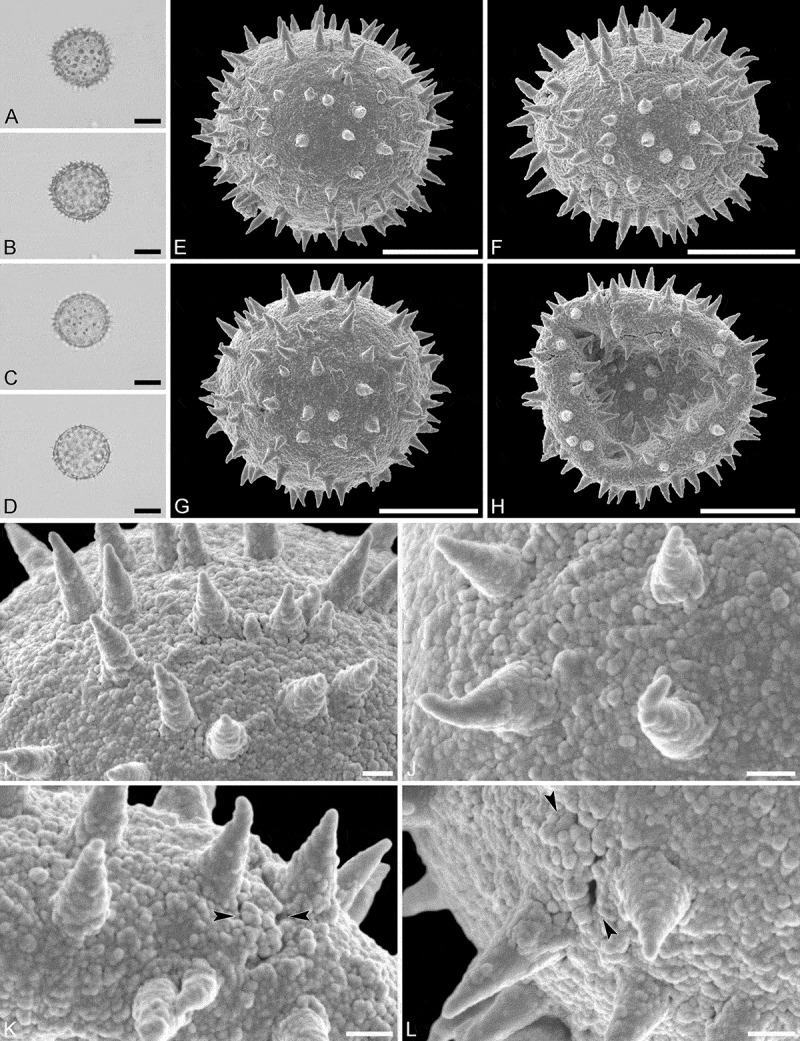
Light microscopy (**A–D**) and scanning electron microscopy (**E–L**) micrographs of *Aristogeitonia lophirifolia* (from Madagascar, coll. Capuron, 23.156-SF [MO]). **A.** Polar view, high focus. **B.** Polar view, optical cross-section. **C.** Equatorial view, high focus. **D.** Equatorial view, optical cross-section. **E.** Polar view. **F.** Polar view. **G.** Oblique polar view. **H.** Infolded grain. **I.** Close-up of interapertural area. **J.** Close-up of interapertural area. **K.** Close-up of aperture, showing membrane (*arrows*). **L.** Close-up of aperture, showing membrane (*arrows*). Scale bars – 10 µm (A–H), 1 µm (I–L).

**Figure 4.**
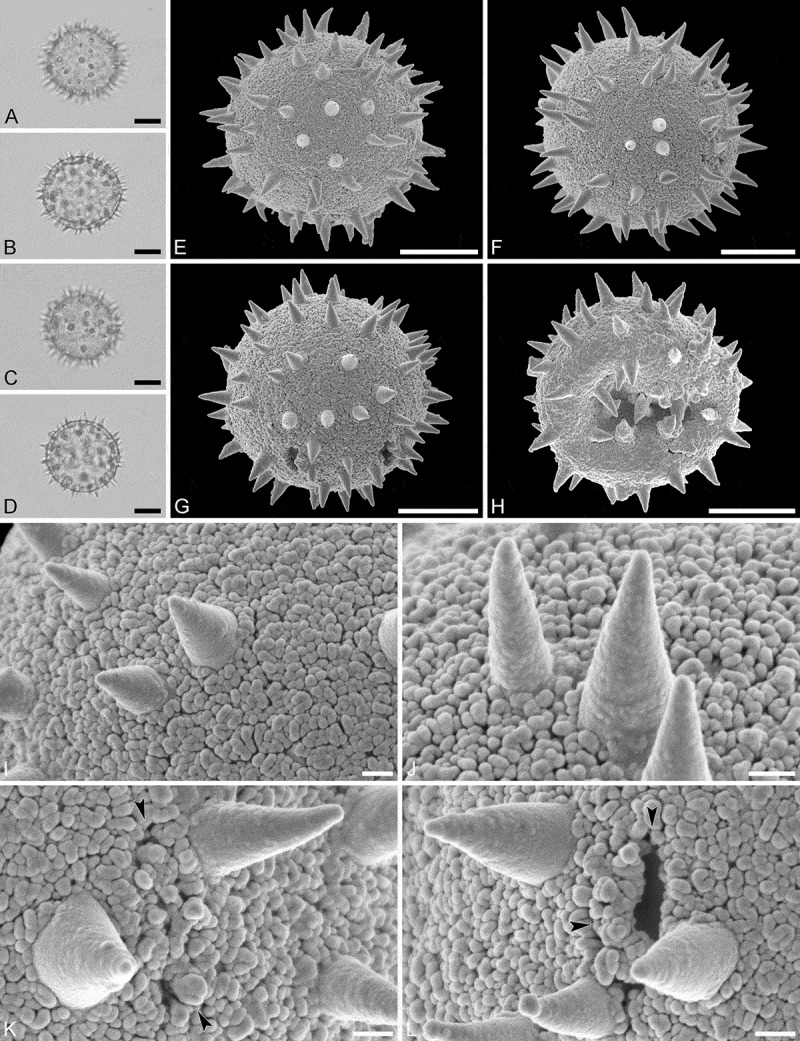
Light microscopy (**A–D**) and scanning electron microscopy (**E–L**) micrographs of *Aristogeitonia monophylla* (from Tanzania, coll. Phillipson, 4954 [MO]). **A.** Polar view, high focus. **B.** Polar view, optical cross-section. **C.** Equatorial view, high focus. **D.** Equatorial view, optical cross-section. **E.** Polar view. **F.** Polar view. **G.** Oblique polar view. **H.** Infolded grain. **I.** Close-up of interapertural area. **J.** Close-up of interapertural area. **K.** Close-up of aperture, showing membrane (*arrows*). **L.** Close-up of aperture, showing membrane (*arrows*). Scale bars – 10 µm (A–H), 1 µm (I–L).

**Figure 5.**
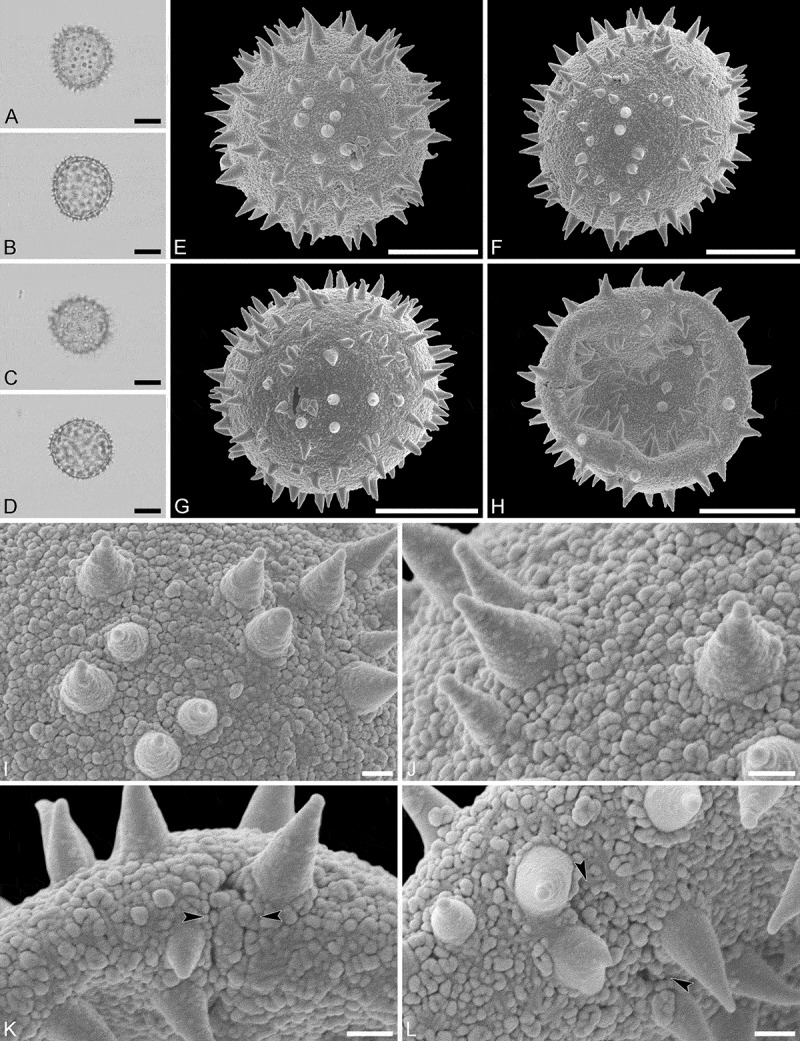
Light microscopy (**A–D**) and scanning electron microscopy (**E–L**) micrographs of *Aristogeitonia perrieri* (from Madagascar, coll. Capuron, 18.462-SF [MO]). **A.** Polar view, high focus. **B.** Polar view, optical cross-section. **C.** Equatorial view, high focus. **D.** Equatorial view, optical cross-section. **E.** Polar view. **F.** Polar view. **G.** Equatorial view. **H.** Infolded grain. **I.** Close-up of interapertural area. **J.** Close-up of interapertural area. **K.** Close-up of aperture, showing membrane (*arrows*). **L.** Close-up of aperture, showing membrane (*arrows*). Scale bars – 10 µm (A–H), 1 µm (I–L).

**Figure 6.**
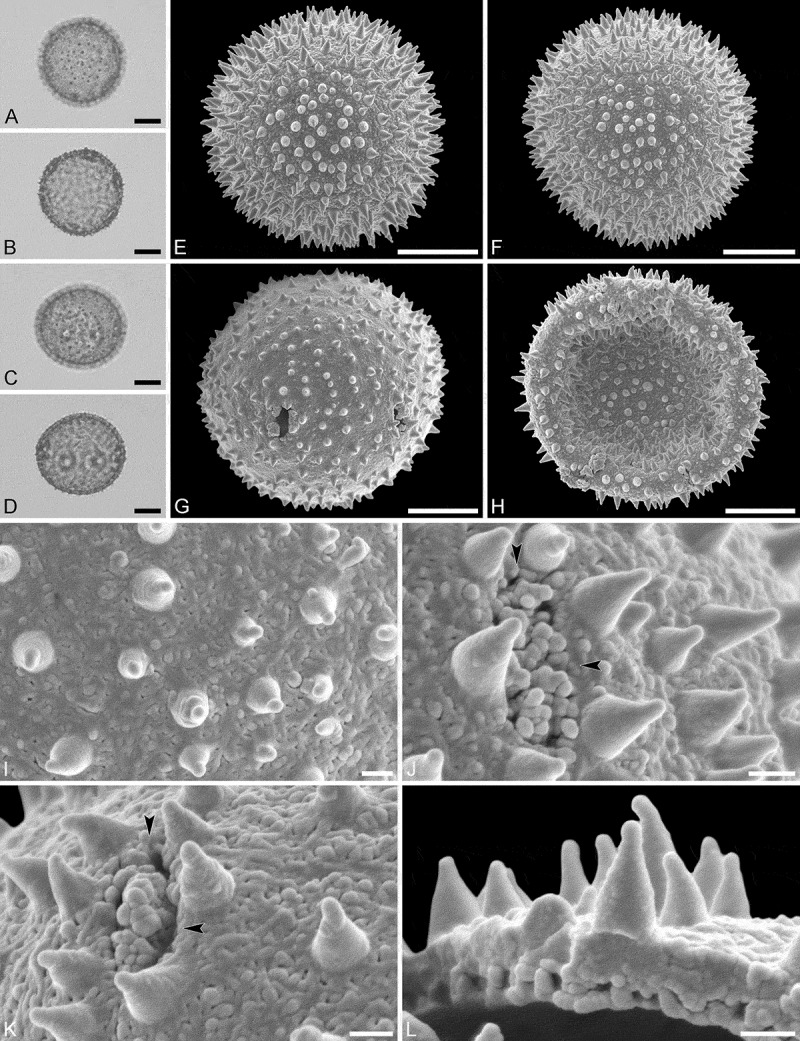
Light microscopy (**A–D**) and scanning electron microscopy (**E–L**) micrographs of *Hyaenanche globosa* (from South Africa, coll. unknown, s.n. [WAG: **E, F, H, J, L**]; from Namibia, coll. Hall, 3912 [PRE: **A**–**D, G, I, K**]). **A.** Polar view, high focus. **B.** Polar view, optical cross-section. **C.** Equatorial view, high focus. **D.** Equatorial view, optical cross-section. **E.** Polar view. **F.** Polar view. **G.** Equatorial view. **H.** Infolded grain. **I.** Close-up of interapertural area. **J.** Close-up of aperture, showing membrane (*arrows*). **K.** Close-up of aperture, showing membrane (*arrows*). **L.** Close-up showing section through pollen wall. Scale bars – 10 µm (A–H), 1 µm (I–L).

**Figure 7.**
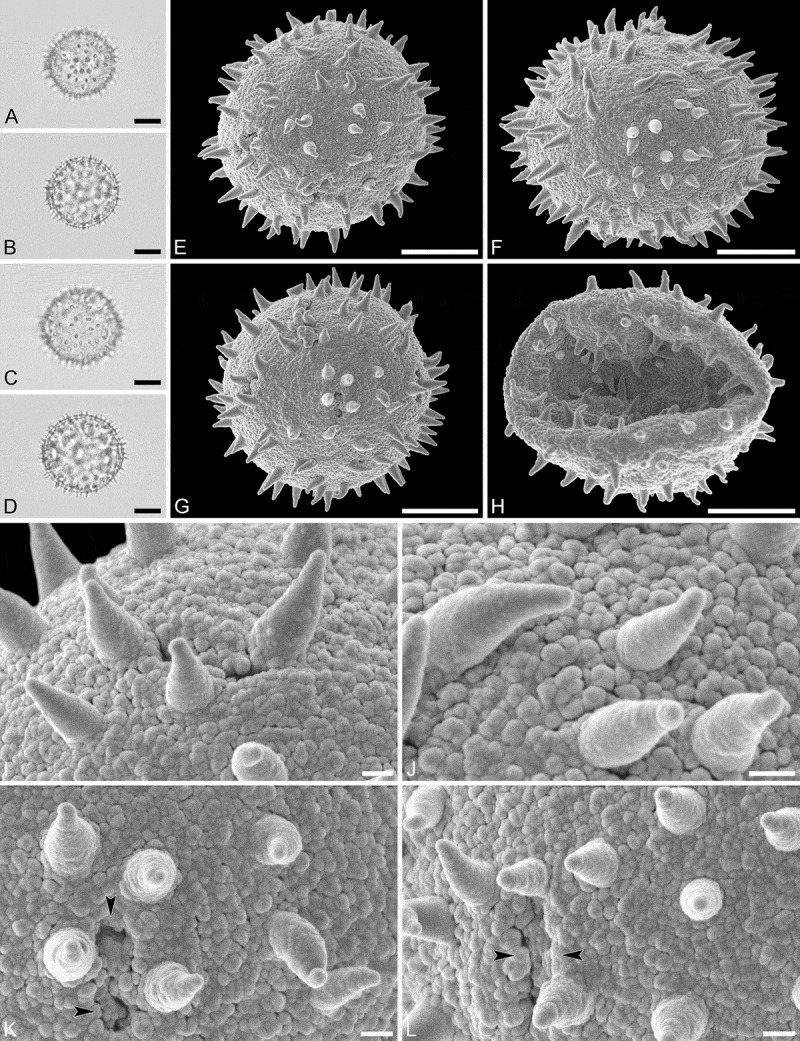
Light microscopy (**A–D**) and scanning electron microscopy (**E–L**) micrographs of *Mischodon zeylanicus* (from India, coll. unknown, s.n. [WAG]). **A.** Polar view, high focus. **B.** Polar view, optical cross-section. **C.** Equatorial view, high focus. **D.** Equatorial view, optical cross section. **E.** Oblique polar view. **F.** Polar view. **G.** Equatorial view. **H.** Infolded grain. **I.** Close-up of interapertural area. **J.** Close-up of interapertural area. **K.** Close-up of aperture, showing membrane (*arrows*). **L.** Close-up of aperture, showing membrane (*arrows*). Scale bars – 10 µm (A–H), 1 µm (I–L).

**Figure 8.**
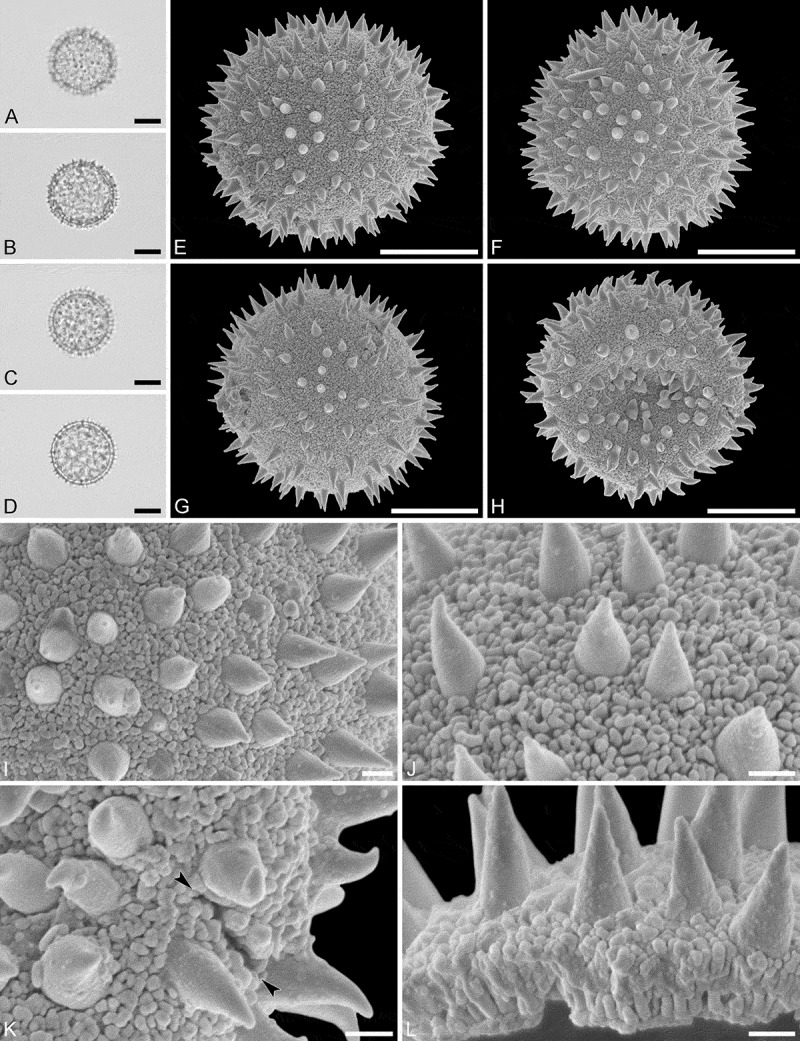
Light microscopy (**A–D**) and scanning electron microscopy (**E–L**) micrographs of *Oldfieldia africana* (from Ivory Coast, coll. Leeuwenberg, 3780 [WAG]). **A.** Polar view, high focus. **B.** Polar view, optical cross-section. **C.** Equatorial view, high focus. **D.** Equatorial view, optical cross-section. **E.** Polar view. **F.** Polar view. **G.** Polar view. **H.** Infolded grain. **I.** Close-up of interapertural area. **J.** Close-up of interapertural area. **K.** Close-up of aperture (*arrows*). **L.** Close-up showing section through pollen wall. Scale bars – 10 µm (A–H), 1 µm (I–L).

**Figure 9.**
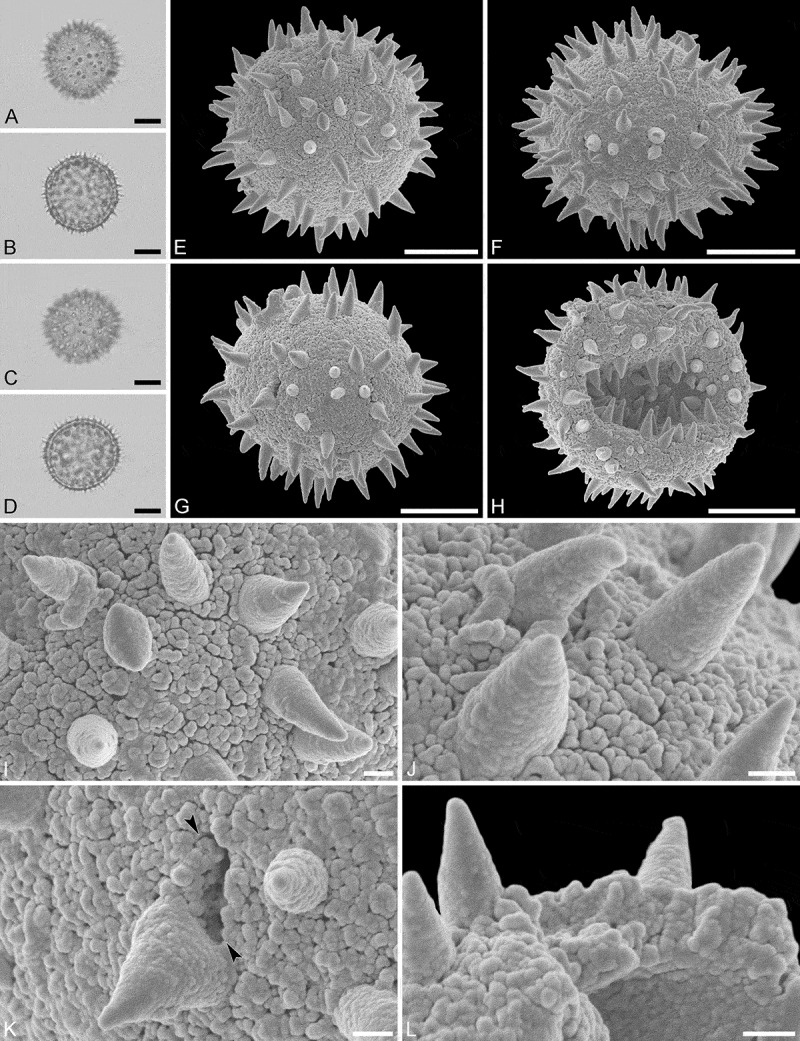
Light microscopy (**A–D**) and scanning electron microscopy (**E–L**) micrographs of *Oldfieldia dactylophylla* (from Tanzania, coll. Meuangulango, 1279 [MO]). **A.** Polar view, high focus. **B.** Polar view, optical cross-section. **C.** Equatorial view, high focus. **D.** Equatorial view, optical cross-section. **E.** Polar view. **F.** Polar view. **G.** Equatorial view. **H.** Infolded grain. **I.** Close-up of interapertural area. **J.** Close-up of interapertural area. **K.** Close-up of aperture, showing membrane (*arrows*). **L.** Close-up showing section through pollen wall. Scale bars – 10 µm (A–H), 1 µm (I–L).

**Figure 10.**
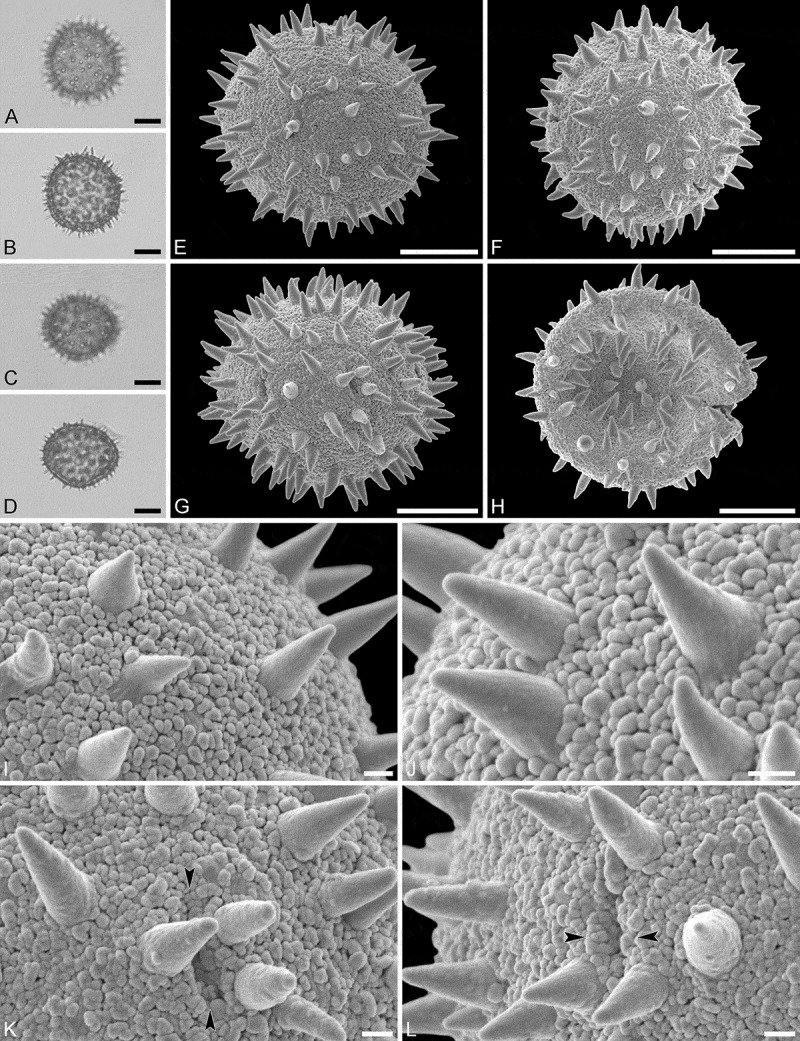
Light microscopy (**A–D**) and scanning electron microscopy (**E–L**) micrographs of *Oldfieldia somalensis* (from Tanzania, coll. Bally, 6880 [EA]). **A.** Polar view, high focus. **B.** Polar view, optical cross-section. **C.** Equatorial view, high focus. **D.** Equatorial view, optical cross-section. **E.** Polar view. **F.** Polar view. **G.** Equatorial view. **H.** Infolded grain. **I.** Close-up of interapertural area. **J.** Close-up of interapertural area. **K.** Close-up of aperture, showing membrane (*arrows*). **L.** Close-up of aperture (*arrows*). Scale bars – 10 µm (A–H), 1 µm (I–L).

**Figure 11.**
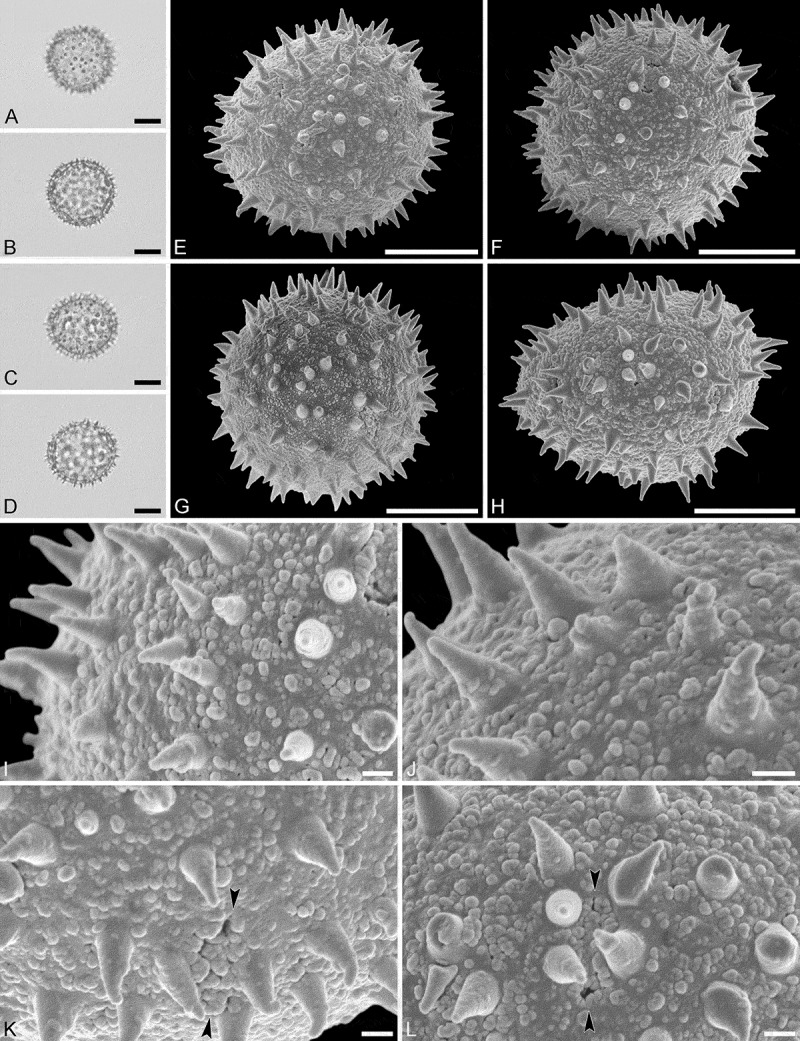
Light microscopy (**A–D**) and scanning electron microscopy (**E–L**) micrographs of *Piranhea longepedunculata* (from Venezuela, coll. Liesner & Gonzalez, 5859 [WAG]). **A.** Polar view, high focus. **B.** Polar view, optical cross-section. **C.** Equatorial view, high focus. **D.** Equatorial view, optical cross-section. **E.** Oblique polar view. **F.** Oblique polar view. **G.** Oblique equatorial view. **H.** Equatorial view. **I.** Close-up of interapertural area. **J.** Close-up of interapertural area. **K.** Close-up of aperture, showing membrane (*arrows*). **L.** Close-up of aperture, showing membrane (*arrows*). Scale bars – 10 µm (A–H), 1 µm (I–L).

**Figure 12.**
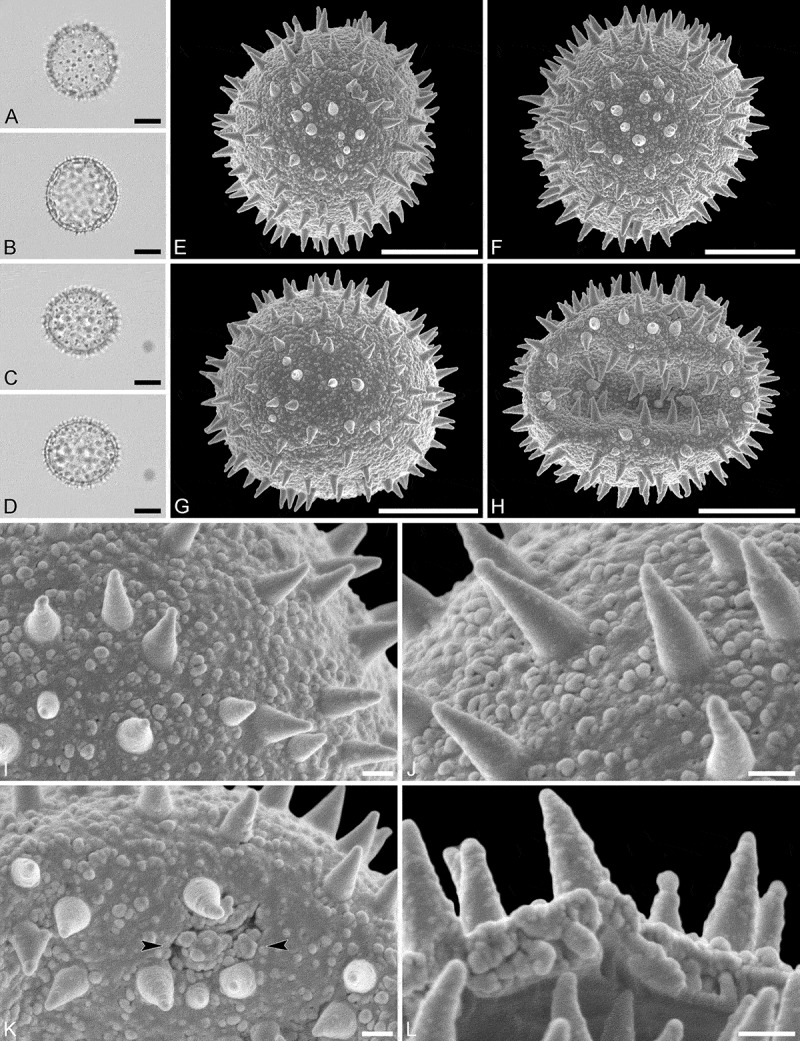
Light microscopy (**A–D**) and scanning electron microscopy (**E–L**) micrographs of *Piranhea trifoliata* (from Brazil, coll. Berg et al., P19790 [WAG]). **A.** Polar view, high focus. **B.** Polar view, optical cross-section. **C.** Equatorial view, high focus. **D.** Equatorial view, optical cross-section. **E.** Polar view. **F.** Polar view. **G.** Polar view. **H.** Infolded grain. **I.** Close-up of interapertural area. **J.** Close-up of interapertural area. **K.** Close-up of aperture, showing membrane (*arrows*). **L.** Close-up showing section through pollen wall. Scale bars – 10 µm (A–H), 1 µm (I–L).

**Figure 13.**
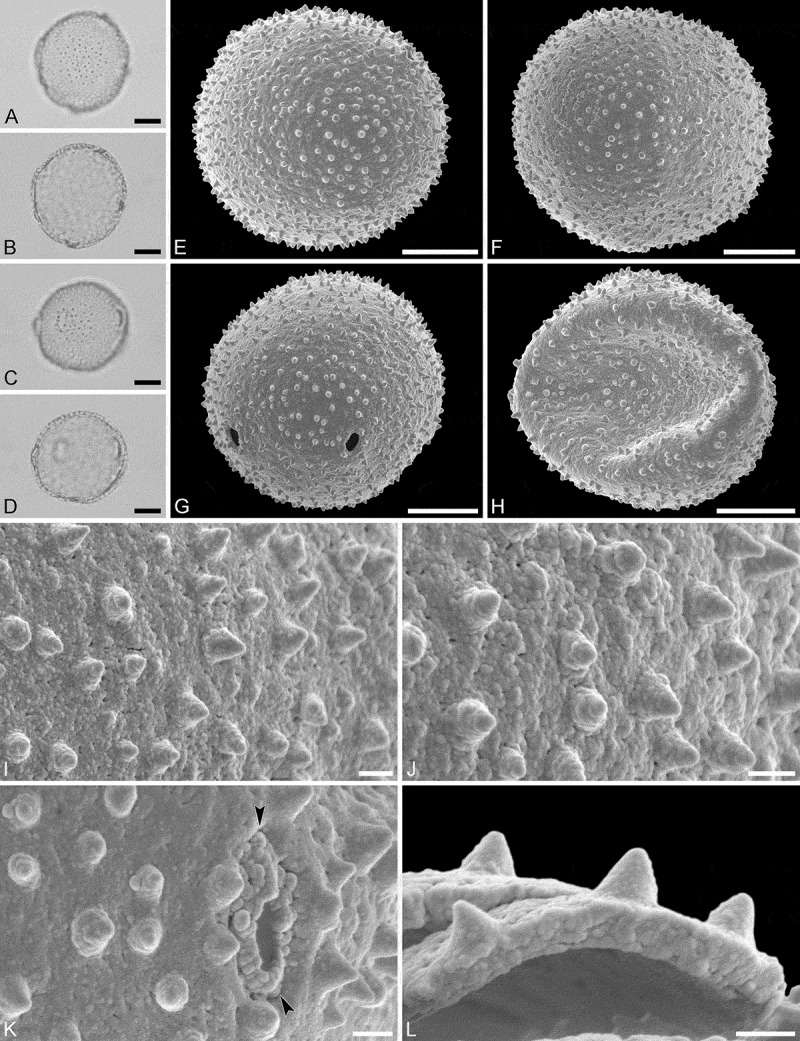
Light microscopy (**A–D**) and scanning electron microscopy (**E–L**) micrographs of *Stachyandra merana* (from Madagascar, coll. Capuron, 23.335-SF [MO]). **A.** Polar view, high focus. **B.** Polar view, optical cross-section. **C.** Equatorial view, high focus. **D.** Equatorial view, optical cross-section. **E.** Polar view. **F.** Polar view. **G.** Equatorial view. **H.** Infolded grain. **I.** Close-up of interapertural area. **J.** Close-up of interapertural area. **K.** Close-up of aperture, showing membrane (*arrows*). **L.** Close-up showing section through pollen wall. Scale bars – 10 µm (A–H), 1 µm (I–L).

**Figure 14.**
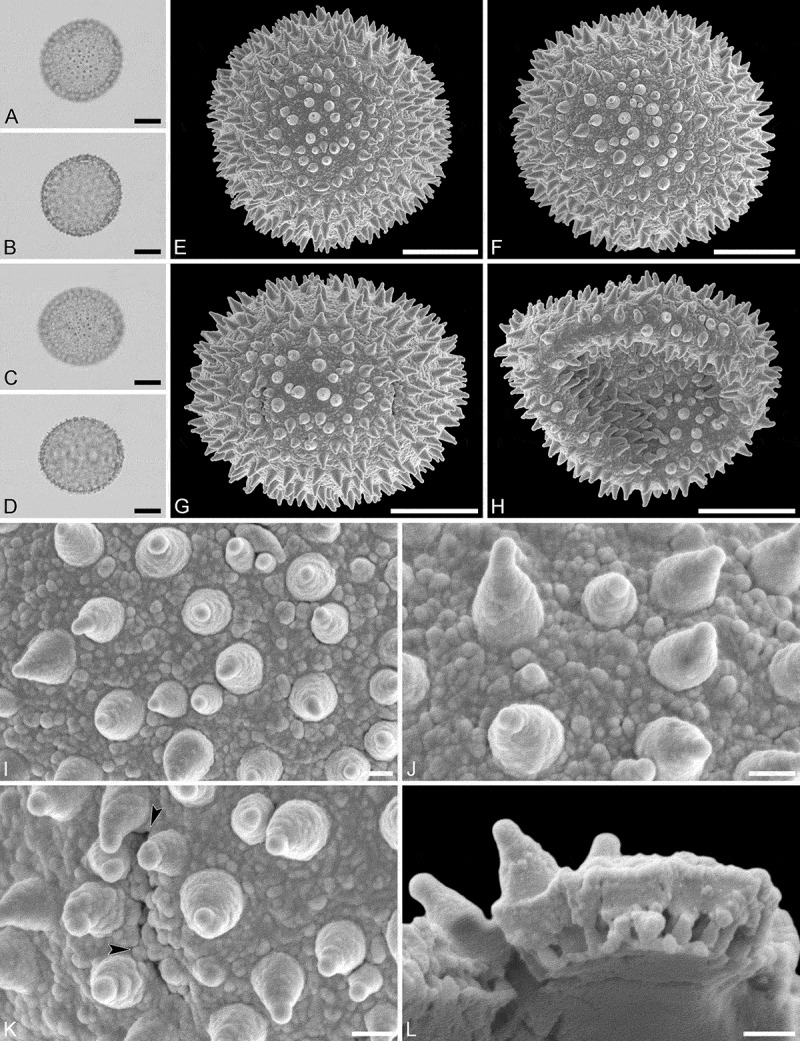
Light microscopy (**A–D**) and scanning electron microscopy (**E–L**) micrographs of *Tetracoccus fasciculatus* (from USA, coll. Langenberg, s.n. [WAG]). **A.** Polar view, high focus. **B.** Polar view, optical cross-section. **C.** Equatorial view, high focus. **D.** Equatorial view, optical cross-section. **E.** Polar view. **F.** Polar view. **G.** Equatorial view. **H.** Infolded grain. **I.** Close-up of interapertural area. **J.** Close-up of interapertural area. **K.** Close-up of aperture, showing membrane (*arrows*). **L.** Close-up showing section through pollen wall. Scale bars – 10 µm (A–H), 1 µm (I–L).

**Figure 15.**
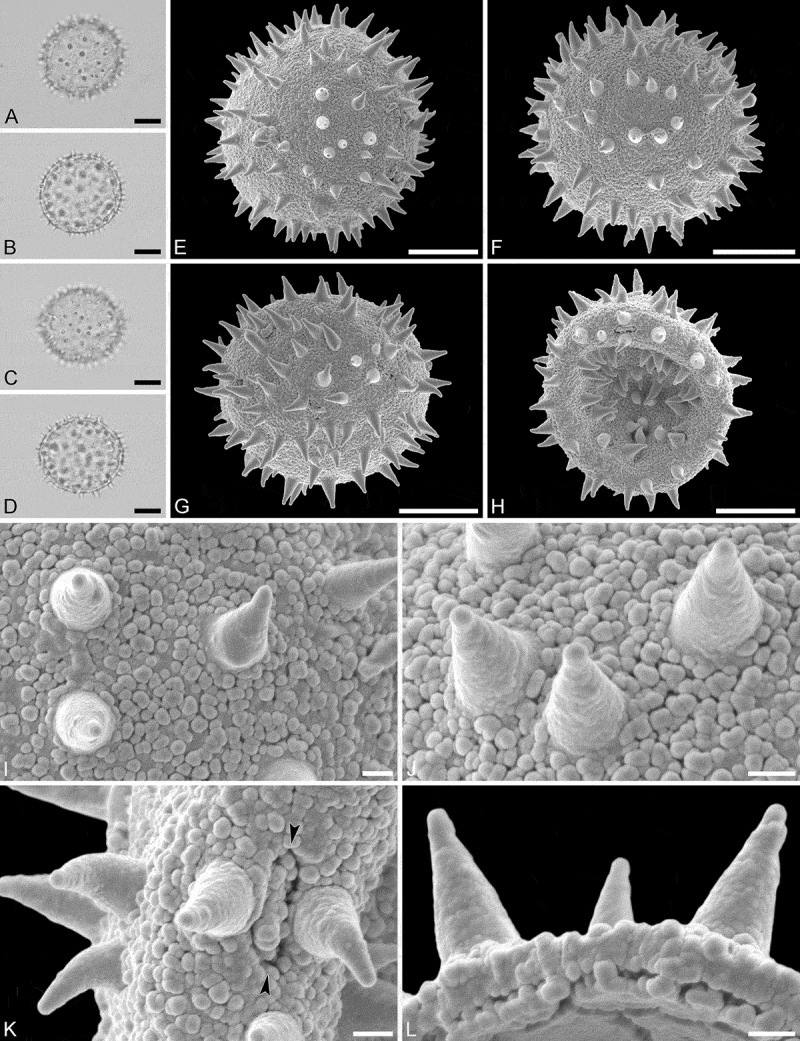
Light microscopy (**A–D**) and scanning electron microscopy (**E–L**) micrographs of *Voatamalo eugenioides* (from Madagascar, coll. Capuron, 22.327-SF [MO]). **A.** Polar view, high focus. **B.** Polar view, optical cross-section. **C.** Equatorial view, high focus. **D.** Equatorial view, optical cross-section. **E.** Polar view. **F.** Polar view. **G.** Equatorial view. **H.** Infolded grain. **I.** Close-up of interapertural area. **J.** Close-up of interapertural area. **K.** Close-up of aperture, showing membrane (*arrows*). **L.** Close-up showing section through pollen wall. Scale bars – 10 µm (A–H), 1 µm (I–L).

**Figure 16.**
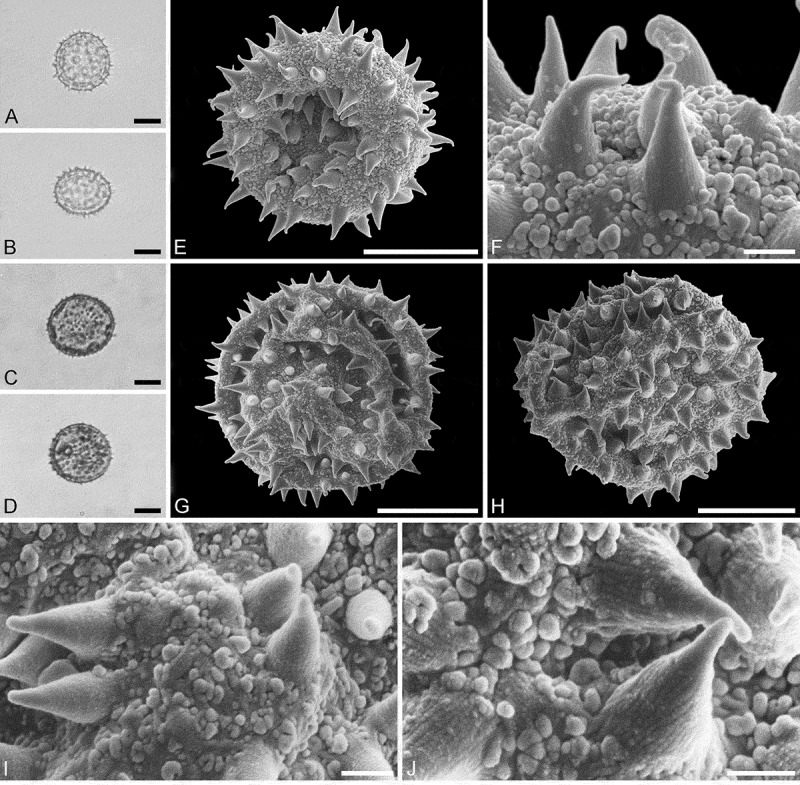
Light microscopy (**A–D**) and scanning electron microscopy (**E–J**) micrographs of the Krappfeld MT (same grain: **A, B, E, F**) and Stolzenbach MT (same grain: **C, D, G, I**; same grain: **H, J**). **A.** Polar view. **B.** Equatorial view. **C.** Polar view. **D.** Equatorial view. **E.** Polar view, infolded grain. **F.** Close-up of interapertural area. **G.** Polar view, infolded grain. **H.** Oblique polar view, infolded grain. **I.** Close-up of interapertural area. **J.** Close-up of interapertural area. Scale bars – 10 µm (A–E, G, H), 1 µm (F, I, J).

**Figure 17.**
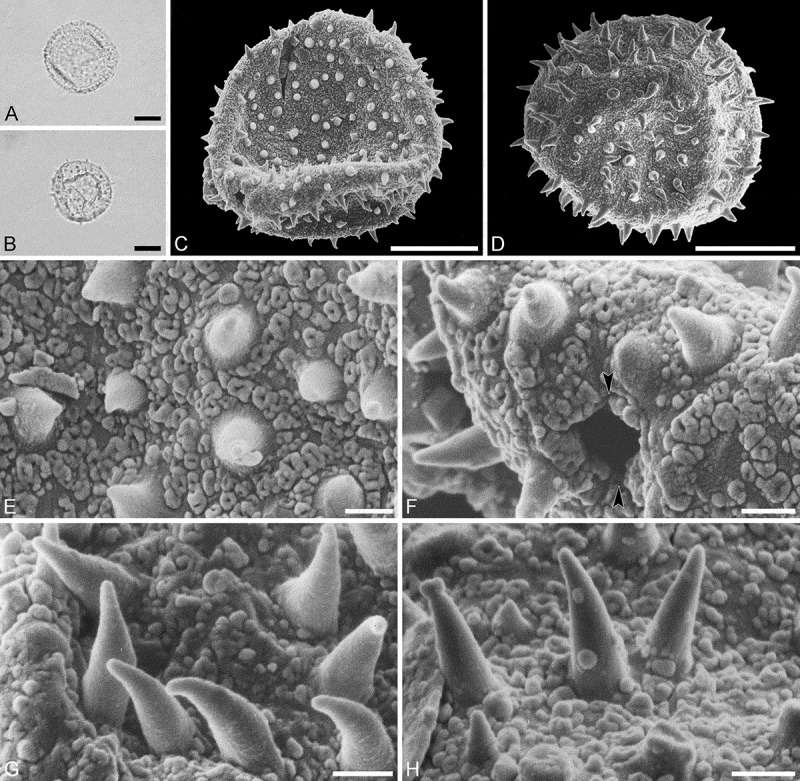
Light microscopy (**A, B**) and scanning electron microscopy (**C–H**) micrographs of the Stolzenbach MT (same grain: **A, C, E**–**G**; same grain: **B, D, H**). **A.** Polar view. **B.** Polar view. **C.** Polar view, infolded grain. **D.** Polar view. **E.** Close-up of interapertural area. **F.** Close-up of aperture (*arrows*). **G.** Close-up of interapertural area. **H.** Close-up of interapertural area. Scale bars – 10 µm (A–D), 1 µm (E–H).

**Figure 18.**
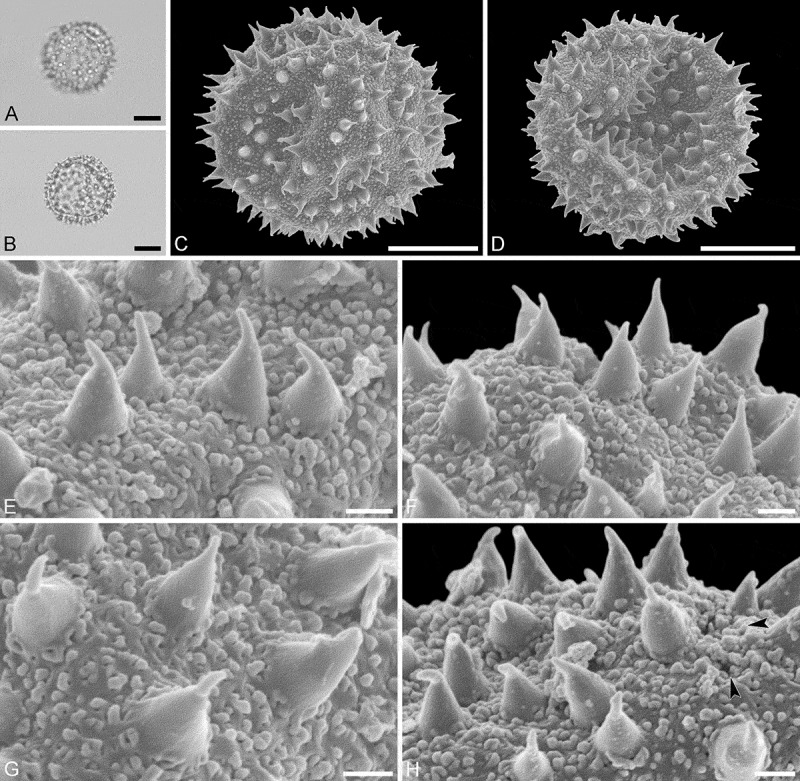
Light microscopy (**A, B**) and scanning electron microscopy (**C–H**) micrographs of the Profen MT (same grain: **A**–**H**). **A.** Polar view, high focus. **B.** Polar view, optical cross-section. **C.** Polar view. **D.** Polar view, opposite site, grain infolded. **E.** Close-up of interapertural area. **F.** Close-up of interapertural area. **G.** Close-up of interapertural area. **H.** Close-up of aperture, showing membrane (*arrows*). Scale bars – 10 µm (A–D), 1 µm (E–H).

**Figure 19.**
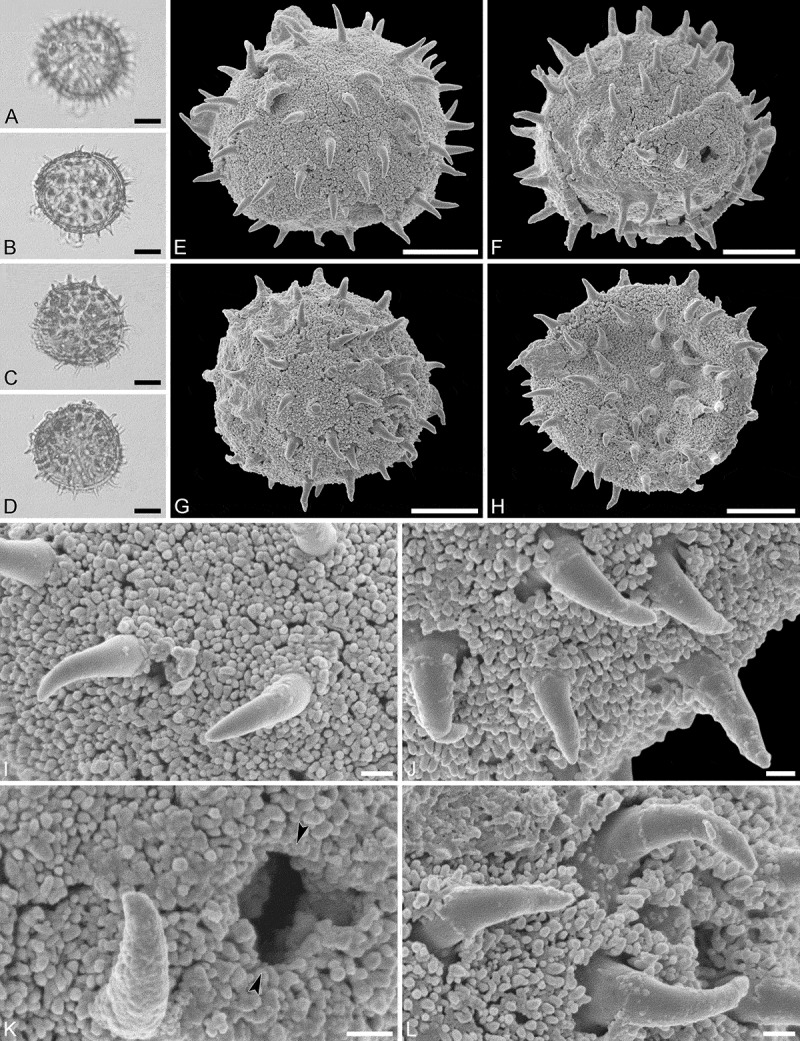
Light microscopy (**A**–**D**) and scanning electron microscopy (**E**–**L**) micrographs of the Mush MT (same grain: **A, B, E, F, I, K**; same grain: **C, D, G, H, J, L**). **A.** Equatorial view, high focus. **B.** Equatorial view, optical cross-section. **C.** Oblique equatorial view. **D.** Oblique equatorial view. **E.** Oblique equatorial view. **F.** Oblique equatorial view, opposite site, grain broken. **G.** Polar view. **H.** Polar view, opposite site, grain infolded. **I.** Close-up of interapertural area. **J.** Close-up of interapertural area. **K.** Close-up of aperture (*arrows*). **L.** Close-up of interapertural area. Scale bars – 10 µm (A–H), 1 µm (I–L).

**Figure 20.**
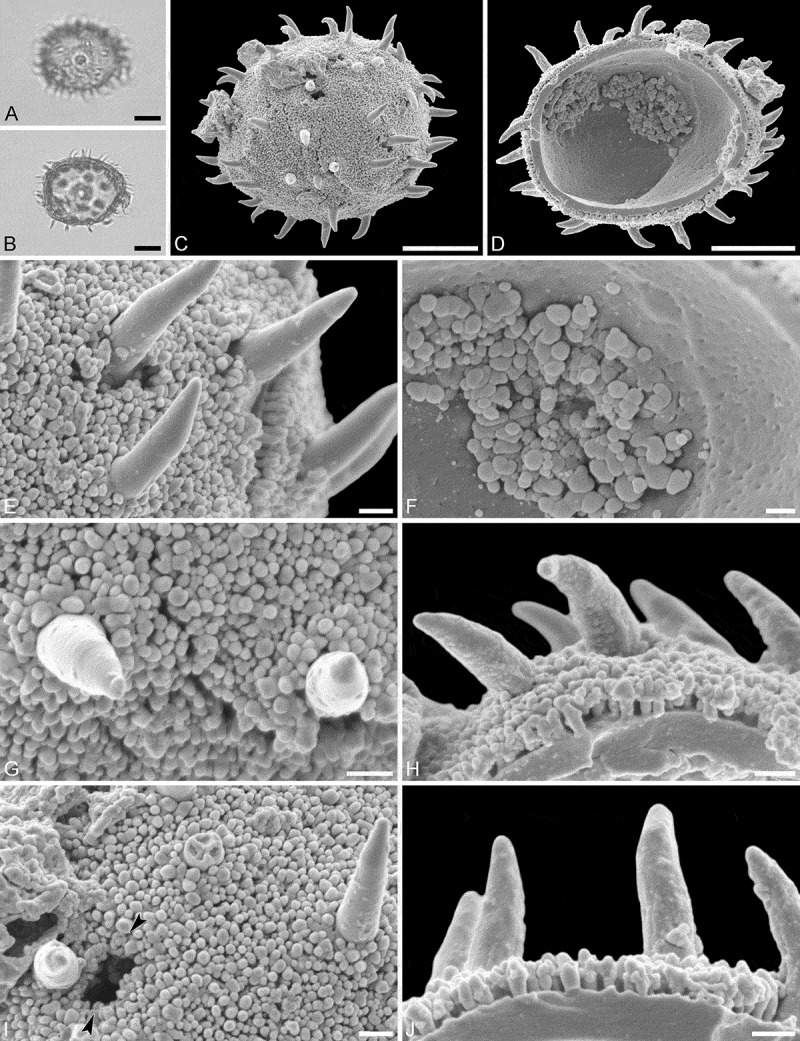
Light microscopy (**A, B**) and scanning electron microscopy (**C**–**J**) micrographs of the Mush MT (same grain: **A–J**). **A.** Equatorial view, high focus. **B.** Equatorial view, optical cross-section. **C.** Oblique equatorial view. **D.** Grain broken, showing the inner side of the pollen. **E.** Close-up of interapertural area. **F.** Close-up showing thickening around aperture on the inner side of pollen wall. **G.** Close-up of interapertural area. **H.** Close-up showing section through pollen wall. **I.** Close-up of aperture (*arrows*). **J.** Close-up showing section through pollen wall. Scale bars – 10 µm (A–D), 1 µm (E–J).

**Figure 21.**
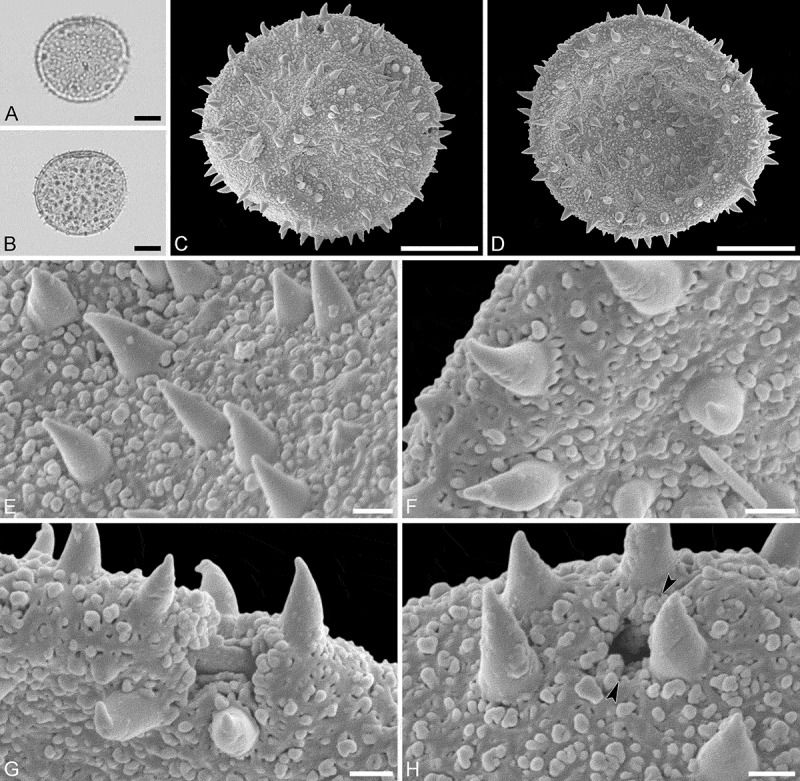
Light microscopy (**A, B**) and scanning electron microscopy (**C–H**) micrographs of the Saldanha MT (same grain: **A**–**H**). **A.** Polar view, high focus. **B.** Polar view, optical cross-section. **C.** Polar view. **D.** Polar view, opposite site, grain infolded. **E.** Close-up of interapertural area. **F.** Close-up of interapertural area. **G.** Close-up of aperture, showing part of membrane (*arrows*). **L.** Close-up of aperture, showing part of membrane (*arrows*). Scale bars – 10 µm (A–D), 1 µm (E–H).

**Figure 22.**
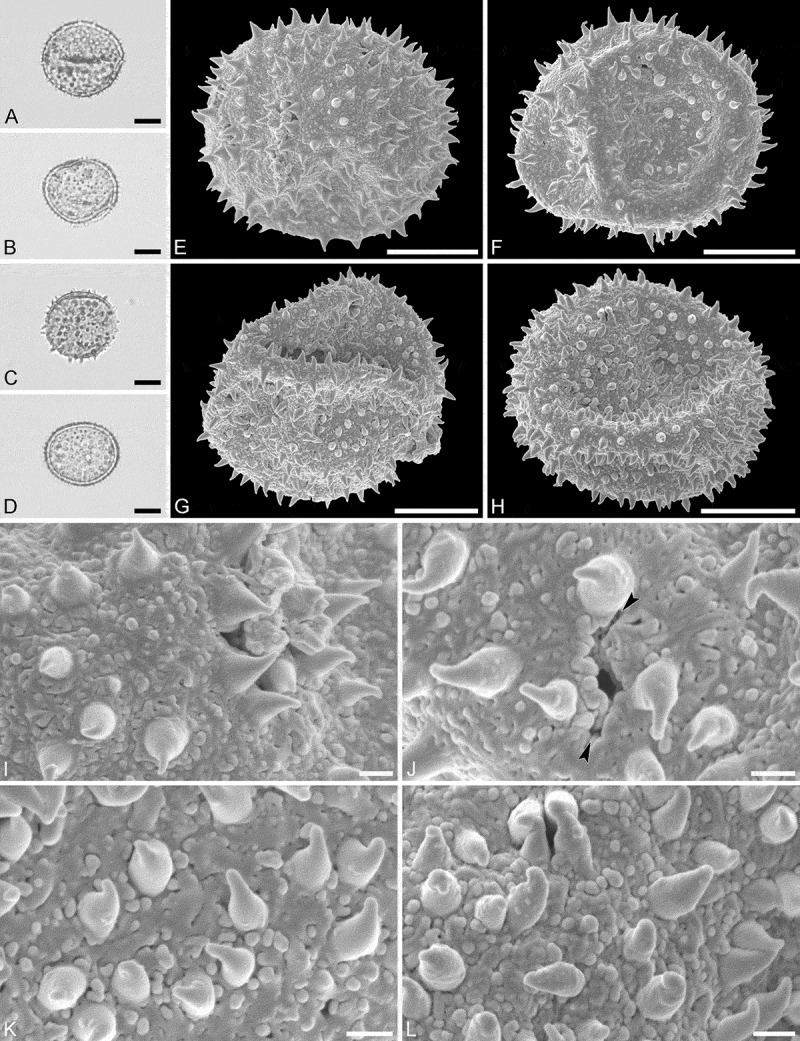
Light microscopy (**A–D**) and scanning electron microscopy (**E–L**) micrographs of the Saldanha MT (same grain: **A, E, I**; same grain **B, F, J**; same grain **C, G, K**; same grain: **D, H, L**). **A.** Polar view. **B.** Oblique polar view. **C.** Oblique equatorial view. **D.** Equatorial view. **E.** Oblique equatorial view. **F.** Oblique equatorial view, infolded grain. **G.** Oblique polar view, folded grain. **H.** Polar view, folded grain. **I.** Close-up of interapertural area. **J.** Close-up of aperture, showing part of membrane (*arrows*). **K.** Close-up of interapertural area. **L.** Close-up of interapertural area. Scale bars – 10 µm (A–H), 1 µm (I–L).

PT 3 occurs in two genera, *Androstachys* (Figure 1) and *Stachyandra* (Figure 13). This pollen is characterised by irregularly placed pori, microechinate sculpture (echini 0.7–1.3 µm high and 30–45 per 100 µm^2^) and nanoechinate aperture membranes (Table V). The pollen grains of the two taxa studied, *Androstachy johnsonii* and *Stachyandra merana*, are so similar that there is no way to distinguish them using LM and/or SEM. The two genera are debatably congeneric (Radcliffe-Smith 2001; Webster 2014).

### Origin, divergence, and dispersal of Afro-Indian Picrodendraceae

Previous molecular studies using fossil age constraints suggested that crown Malpighiales began to radiate in the late Early Cretaceous (middle Aptian to middle Albian), at 119.4–110.7 Ma (Davis et al. 2005) or 113.1–106.1 Ma (Xi et al. 2012) (but see also Wikström et al. 2001; Magallón & Castillo 2009; Bell et al. 2010). So far, estimates for the age of the Phyllanthaceae/Picrodendraceae clade fall in the latest Early Cretaceous (Albian), at 114–105.8 Ma (Davis et al. 2005) or in the early Late Cretaceous (Cenomanian to Coniacian), at 101–86.5 Ma (Xi et al. 2012). Estimates for the age of crown-group Picrodendraceae suggest a middle Late Cretaceous (Turonian to Campanian) origin at 92.9–72 Ma (Xi et al. 2012).

Since all the Afro-Indian Picrodendraceae form a monophyletic clade (Figures 24, 25; Wurdack 2008; Wurdack & Davis 2009), it is clear they descended from a single common ancestor that at some point dispersed into Africa. The molecular phylograms of Wurdack and Davis (2009), and Xi et al. (2012), with seven genera, place the South American *Podocalyx *as sister to the other six genera of the Picrodendraceae that were sampled, which form a grade from *Tetracoccus* to *Austrobuxus*. The bipartition network presented here (Figure 24) suggests two major clades, one including all American and African taxa, and the other including all Australasian taxa, with a potential divergence (or origin) point to be placed somewhere close to *Podocalyx*.

**Figure 23.**
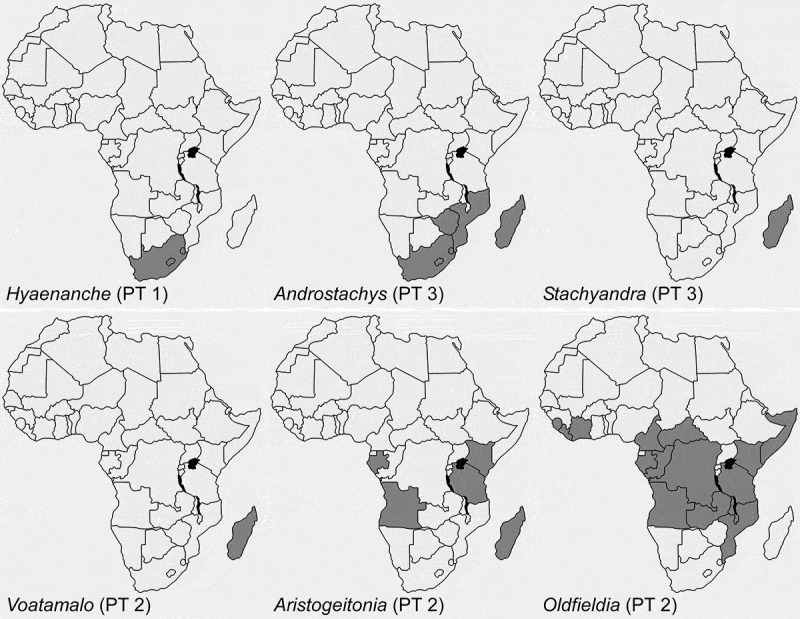
Maps showing the African countries (dark grey) where Picrodendraceae have been reported as part of the modern flora. The pollen types (PT 1–PT 3) are noted.

**Figure 24.**
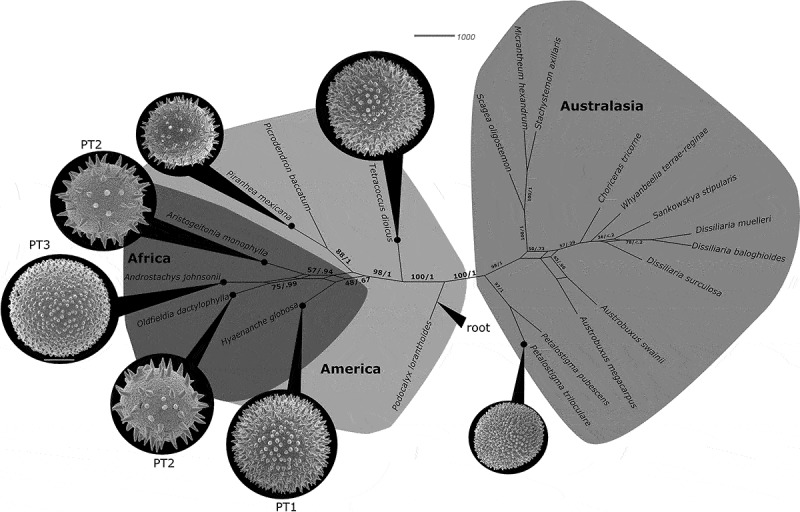
Consensus network based on ML bootstrap replicate summarised with a 20% cutoff. Support values for the split are shown as ML bootstrap/BI posterior probability. Pollen types of different taxa are illustrated at the same scale (scale bar = 10 µm). Geography of the different clades are indicated by different background colour.

It is interesting that PT 1, found in the African *Hyaenanche*, is extremely similar to pollen occurring in the early branching *Tetracoccus* (second branching American lineage; Figure 24) and in *Piranhea*, that is part of the sister clade to the Afro-Indian taxa. PT 1 is clearly a basal or primitive (plesiomorphic) pollen occurring in early diverging/branching Picrodendraceae. This suggests that PT 1 is the ‘original’ pollen type of the lineage that dispersed into Africa from the Americas; therefore PT 1 is ancestral to PT 2, a pollen type occurring in most of the extant Afro-Indian taxa (*Aristogeitonia, Mischodon, Oldfieldia* and *Voatamalo*) and showing the widest extant distribution (Figure 23). Lineages with PT 2 subsequently led to the evolution of PT 3, occurring in both *Androstachys* and *Stachyandra*.

Picrodendraceae bear mostly explosive dehiscent schizocarp (capsular) fruits (*Androstachys, Aristogeitonia, Austrobuxus, Choriceras, Dissiliaria, Hyaenanche, Kairothamnus, Longetia, Micrantheum, Mischodon, Neoroepera, Paradiodendron, Piranhea, Podocalyx, Pseudanthus, Sankowskya, Scagea, Stachyandra, Stachystemon, Tetracoccus, Voatamalo, Whyanbeelia*) resulting in very restricted autochorous dispersal (up to a few metres), believed to be primitive in the family (Webster 1994a, 2014). About half of the genera produce carunculate seeds (*Androstachys, Austrobuxus, Hyaenanche, Longetia, Micrantheum, Neoroepera, Oldfieldia, Petalostigma, Pseudanthus, Sankowskya, Scagea, Stachyandra, Stachystemon, Tetracoccus, Voatamalo, Whyanbeelia*) that are dispersed by ants (myrmecochory; Webster 1994a, 2014). Only three genera have fleshy or drupaceous seeds/fruits (*Oldfieldia, Petalostigma, Picrodendron*) that are primarily dispersed by birds and/or mammals (endozoochory/ornithochory), a trait believed to be derived in the family (Webster 1994a, 2014). The African *Oldfieldia* are dispersed by mammals, mostly primates (Koné et al. 2008), the Australian *Petalostigma* by emus (Clifford & Monteith 1989), and the West Indies *Picrodendron* by birds and iguanas (Alberts 1999; Hines 2016). According to Webster (1994a, 2014) the basal Phyllanthaceae, Picrodendraceae and Euphorbiaceae typically bear capsular fruits with dry seed-coats excluding long distance dispersal across wide ocean barriers. The predominant autochorous and myrmecochorous dispersal of basal Picrodendraceae and their current distribution suggests they could have dispersed across the Southern Hemisphere prior to later stages of the Gondwana breakup when ocean barriers became too wide.

The Gondwana landmass started to fragment during the Early to Middle Jurassic, at 180–160 Ma (summarised by: e.g. McLoughlin 2001; Chatterjee et al. 2013; Torsvik & Cocks 2013). In the Early Cretaceous, at ~130 Ma (Barremian), Gondwana started to separate into two approximately equal halves, (1) West Gondwana composed of South America and Africa, and (2) East Gondwana composed of Madagascar, India, Antarctica and Australia. Contemporaneously, Madagascar/India started to drift apart from Antarctica/Australia. This scenario continued until *c*. 90 Ma, when India started to break away from Madagascar, and India then continued its northward journey towards Asia. From that time on Madagascar became part of the African plate (Chatterjee et al. 2013). Also, eastern South America and west/central Africa were closely aligned until 90–80 Ma (Gaina et al. 2013), with potential pathways for plant dispersal between the two continents. Furthermore, tectonic models of East Gondwana (Gibbons et al. 2013) suggest that Africa, Madagascar and India were close until 80–70 Ma, allowing for plant dispersal among these landmasses until at least the latest Upper Cretaceous (Campanian/Maastrichtian). Additionally, paleogeographic reconstructions and terrestrial fossil records suggest that southern South America and West Antarctica were connected by a land bridge (Weddellian Isthmus) believed to have been functional until the late Paleocene (Reguero et al. 2014, terrestrial vertebrates) or the middle Eocene (Ghiglione et al. 2013, terrestrial vertebrates), or even the early Oligocene (Graham 2018b, plants). Based on the paleogeography and taking into account dispersal mechanisms of the family it is clear that if Picrodendraceae dispersed across a southern route into Africa they must have done so prior to the end Cretaceous. Also, since *Mischodon* has an advanced pollen type (PT 2) and is nested within the African clade (Wurdack 2008) that scenario suggests they must have dispersed from Africa/Madagascar into India in the latest Cretaceous (*c*. 80–70 Ma; Gibbson et al. 2013) or the early Cenozoic. But that does not fit with the fossil record of the family.

Fossil records of the Phyllanthaceae/Picrodendraceae clade can be traced back to the Upper Cretaceous. Webster (2014) summarised the macrofossil record of Euphorbiaceae s.l. citing fossil woods, fruits and inflorescences. However, neither Webster nor previous authors affiliate any macrofossil directly to the Picrodendraceae (or Oldfieldioideae of the Euphorbiaceae s.l.). Long before, Metcalfe and Chalk (1950) presented a clear overview of the wood anatomy of Euphorbiaceae s.l., in which they recognised three groups within the so-called ‘Phyllanthoideae’ (= today’s Phyllanthaceae and Picrodendraceae): Group A (*Aporosa* or *Aporusa* Type), Group B (*Glochidion* Type) and ‘Other genera of the Phyllanthoideae’, which mainly represent Picrodendraceae (Hayden 1994). Many fossil wood genera (*Paraphyllanthoxylon, Phyllanthinium, Glochidioxylon, Bridelioxylon* [syn. *Bischofioxylon*], *Securinegoxylon* and *Bischofinium*) have been proposed to accommodate the ‘Phyllanthoideae’ *sensu* Metcalfe and Chalk (1950; see also Mädel 1962; Prakash et al. 1986). Of these, only *Paraphyllanthoxylon* (taxa of species group B *sensu* Herendeen 1991) with numerous uniseriate rays and longer marginal rows, or more pronounced ray heterogeneity, can be labelled as ‘phyllanthoid’. *Paraphyllanthoxylon* is a very broadly defined fossil genus with numerous species of various ages (Upper Cretaceous to late Cainozoic) described from many parts of the world (for a summary see Gryc et al. 2009; Nunes et al. 2018). The oldest fossil wood record of Phyllanthaceae is represented by the type species of *Paraphyllanthoxylon, Paraphyllanthoxylon arizonense* Bailey, from the Cenomanian of Arizona (Bailey 1924).

The worldwide Cretaceous ‘Phyllanthoideae’ wood records are accompanied by Phyllanthaceae fruit/seeds from the late Maastrichtian of India (Kapgate et al. 2017). The earliest macrofossils showing morphology of modern Picrodendraceae (Table VII) are inflorescences and flowers from the late Paleocene of Tennessee (as *Protoarecoidea buchananensis*; Feldman 1990). These flowers also contain *in situ* pollen that represent the earliest known pollen records of this family, along with dispersed pollen described from the late Paleocene of southeast Australia (Harris 1965; Stover & Partridge 1973). Following the Paleocene, fossil Picrodendraceae pollen (Table VII) are known from the Eocene of Europe (this study; Zetter & Hofmann 2008; Hofmann et al. 2011; Zetter et al. 2011), the Americas (Tschudy & van Loenen 1970; Tschudy 1973; Graham et al. 2000), Australasia (Stover & Partridge 1973) and Antarctica (Truswell & Macphail 2009).

**Table VII.** Fossil record of Picrodendraceae.

Taxon	Organ(s)	Affinity	Locality	Geography	Continent	Period/epoch/stage	Age (ICS 2017)	Range	References
*Protoarecoidea buchananensis*	Inflorescence, flowers, pollen (LM, SEM, TEM)	*Tetracoccus, Piranhea, Picrodendron*	Buchanan, Tennessee	USA	North America	Late Paleocene (Thanetian)	59.2–56.0		Feldman 1990
*Echiperiporites*	Pollen (LM)	*Picrodendron*	Mississippi embayment area	USA	North America	Early Eocene (Ypresian)	59.2–56.0		Tschudy & van Loenen 1970; Tschudy 1973
*Malvacipollis tschudyi*	Pollen (LM)	*Picrodendron*	Saramaguacán Formation	Cuba	North America	Middle Eocene (Lutetian-Bartonian)	47.8–37.8		Graham et al. 2000
*Aristogeitonia* type	Pollen (LM, SEM)	*Piranhea, Hyaenanche*	St Pankraz, Salzburg	Austria	Europe	Early Eocene (Ypresian)	56–47.8		Hofmann et al. 2011
Krappfeld MT	Pollen (LM, SEM)	*Piranhea, Hyaenanche*	Krappfeld	Austria	Europe	Early Eocene (Ypresian)	56–47.8		This study
Stolzenbach MT	Pollen (LM, SEM)	*Piranhea, Hyaenanche*	Stolzenbach	Germany	Europe	Middle Eocene (Lutetian)	47.8–41.2		This study
*Multiporopollenites; Nothofagidites* sp. aff. *N. echinata*	Pollen (LM)	*Piranhea, Hyaenanche*	Helmstedt, Wulfersdorfer Flözgruppe	Germany	Europe	Middle Eocene (Lutetian)	47.8–41.2		Lenz 2000, 2005
Profen MT	Pollen (LM, SEM)	*Piranhea, Hyaenanche*	Profen	Germany	Europe	Middle Eocene (Bartonian)	41.2–37.8		This study
Saldanha MT	Pollen (LM, SEM)	*Hyaenanche*	Saldanha Bay	South Africa	Africa	Early Miocene (Aquitanian-Burdigalian)	23.03–15.97		This study
Mush MT	Pollen (LM, SEM)	*Aristogeitonia, Mischodon, Oldfieldia, Voatamalo*	Mush Valley	Ethiopia	Africa	Early Miocene (Aquitanian)	22.63–21.73 (absolute dating)		This study
*Malvacipollis diversus*	Pollen (LM)	*Austrobuxus, Dissiliaria*	Gippsland Basin	SE Australia	Australasia	Late Paleocene (Thanetian)	59.2–56.0	Late Paleocene through early Eocene	Stover & Partridge 1973
*Malvacipollis diversus*	Pollen (LM)	*Austrobuxus, Dissiliaria*	Princetown area, Victoria	SE Australia	Australasia	Late Paleocene (Thanetian)	59.2–56.0		Harris 1965
*Malvacipollis subtilis*	Pollen (LM)	*Austrobuxus, Dissiliaria*	Gippsland Basin	SE Australia	Australasia	Early Eocene (Ypresian)	56–47.8	Early Eocene through late Miocene	Stover & Partridge 1973
*Malvacipollis diversus*	Pollen (LM)	*Austrobuxus, Dissiliaria*	New South Wales	E Australia	Australasia	Early Eocene (Ypresian)	56–47.8	Early Eocene through Pliocene	Martin 1974
*Plagianthus*	Pollen (LM)	*Austrobuxus, Dissiliaria*	Dannevirke Series	New Zealand	Australasia	Early Eocene (Ypresian)	56–47.8	Early Eocene through Holocene	Couper 1960
*Malvacipollis diversus*	Pollen (LM)	*Austrobuxus, Dissiliaria*	One Tree Hill, St Vincent Basin	S Australia	Australasia	Middle Eocene (Lutetian-Bartonian)	47.8–37.8		Alley & Broadbridge 1992
*Malvacipollis diversus*	Pollen (LM)	*Austrobuxus, Dissiliaria*	Murray Basin	SE Australia	Australasia	Middle Eocene (Bartonian)	41.2–37.8	Middle Eocene through late Eocene	Macphail 1999
*Malvacipollis subtilis*	Pollen (LM)	*Austrobuxus, Dissiliaria*	Murray Basin	SE Australia	Australasia	Middle Eocene (Bartonian)	41.2–37.8	Middle Eocene through Pliocene	Macphail 1999
*Malvacipollis diversus*	Pollen (LM)	*Austrobuxus, Dissiliaria*	Werillup Formation	W Australia	Australasia	Middle Eocene (Bartonian)	41.2–37.8	Late middle Eocene through early late Eocene	Hos 1975; Stover & Partridge 1982
*Malvacipollis diversus*	Pollen (LM)	*Austrobuxus, Dissiliaria*	Prydz Bay	E Antarctica	Antarctica	Late Eocene (Priabonian)	37.8–33.9		Truswell & Macphail 2009
*Malvacipollis subtilis*	Pollen (LM)	*Austrobuxus, Dissiliaria*	Prydz Bay	E Antarctica	Antarctica	Late Eocene (Priabonian)	37.8–33.9		Truswell & Macphail 2009
*Malvacipollis* (*Micrantheum*) *spinyspora*	Pollen (LM)	*Micrantheum, Neoroepera*	Manuherikia Group, Central Otago	New Zealand	Australasia	Early Miocene (Aquitanian-Burdigalian)	23.03–15.97	Early Miocene through Pliocene	Mildenhall 1989
*Malvacipollis* (*Micrantheum*) *spinyspora*	Pollen (LM)	*Micrantheum, Neoroepera*	Murray Basin	SE Australia	Australasia	Late Miocene (Tortonian-Messinian)	11.63–5.33	Late Miocene through Pliocene	Knight & Martin 1989; Mcphail & Truswell 1993
*Malvacipollis* (*Micrantheum*) *spinyspora*	Pollen (LM)	*Micrantheum, Neoroepera*	New South Wales	E Australia	Australasia	Pliocene (Zanclean-Piacenzian)	5.333–2.58	Pliocene through Pleistocene	Martin 1974
*Helciporites astrus*	Pollen (LM)	*Longetia*	Gippsland Basin	SE Australia	Australasia	Early Eocene (Ypresian)	56.0–47.8	Early Eocene through late Eocene	Stover & Partridge 1973
*Polyorificites oblatus*	Pollen (LM)	*Longetia*	New South Wales	E Australia	Australasia	Late Eocene (Priabonian)	37.8–33.9	Late Eocene through Miocene	Martin 1974

The fossil record supports an American origin of Picrodendraceae (Table VII). The fossils at hand suggest that the family was well established in the Americas during the late Paleocene. The fossil record also supports a southern dispersal route, from South America via Antarctica into Australasia, during the late Paleocene, a migration route believed to have been open for plant dispersal during most of the Paleogene (Ghiglione et al. 2013; Reguero et al. 2014; Graham 2018a, 2018b). There are, however, no fossil records from Africa, Madagascar or India from the late Cretaceous or Paleocene to demonstrate the presence of Picrodendraceae in this part of the world during that time. Thus, the European Eocene Krappfeld, Stolzenbach and the Profen fossil pollen MTs become important as the only known representatives of an extinct early diverging lineage of the American-Afro-Indian clade, positioned close to *Piranhea*. The current Picrodendraceae fossil records indicate the family dispersed into Africa from Europe via a second dispersal route, namely from the Americas via the North Atlantic Land bridge during periods of elevated temperatures in the Paleocene and/or Eocene. The North Atlantic Land Bridge is known as a gateway for numerous thermophilic plants, even some of today’s tropical to subtropical plant groups, that were able to disperse freely between the Americas (Greenland) and Europe until the late Eocene (e.g. Tiffney 1985, 2000; Grímsson et al. 2018a). More cold tolerant plants were able to follow this route until the middle to late Miocene (Denk et al. 2010, 2011; Graham 2018a, 2018b). Such a ‘northern route’ has recently been proposed for another predominantly southern hemispheric group, the Loranthaceae, believed to have conquered Africa from Asia during the Eocene (Grímsson et al. 2017b, 2018c). During the Eocene, Europe was influenced by hot and humid climate (Zachos et al. 2001; Mosbrugger et al. 2005), comparable to that of present day tropics to subtropics. In Europe, this paleoclimate sustained a special thermophilic flora, so-called Paratropical Rainforest (e.g. Mai 1995), that disappeared at the end of the Eocene. With tropical and subtropical climate equivalents and corresponding vegetation reaching from the equator towards mid latitudes and even high latitudes, temperatures would not have been a barrier to a southward dispersal of Picrodendraceae from Europe into Africa during the Eocene. The African fossil records show that Picrodendraceae had diverged into at least two different lineages prior to the early Miocene. This is evident by the early Miocene Saldanha MT pollen, that is clearly of the extant PT 1 lineage, and the early Miocene Mush MT, that represents an extinct ancestral taxon of the extant PT 2 lineage. Studies by some of the present authors on late Oligocene to early Miocene palynofloras from Ethiopia and South Africa have yielded no additional fossil pollen that truly represent the extant PT 2 pollen. The only pollen discovered so far are the primitive basal PT 1 from Saldanha Bay (South Africa) and the extinct ancestral type to the PT 2 lineage from Mush (Ethiopia). Therefore, the current fossil data suggests that both the extant PT 2 and PT 3 pollen had not evolved during the latest Oligocene or the earliest Miocene, but are post earliest Miocene taxa. Still, this could easily change in the future as more African palynofloras are studied and screened for Picrodendraceae pollen.

**Figure 25.**
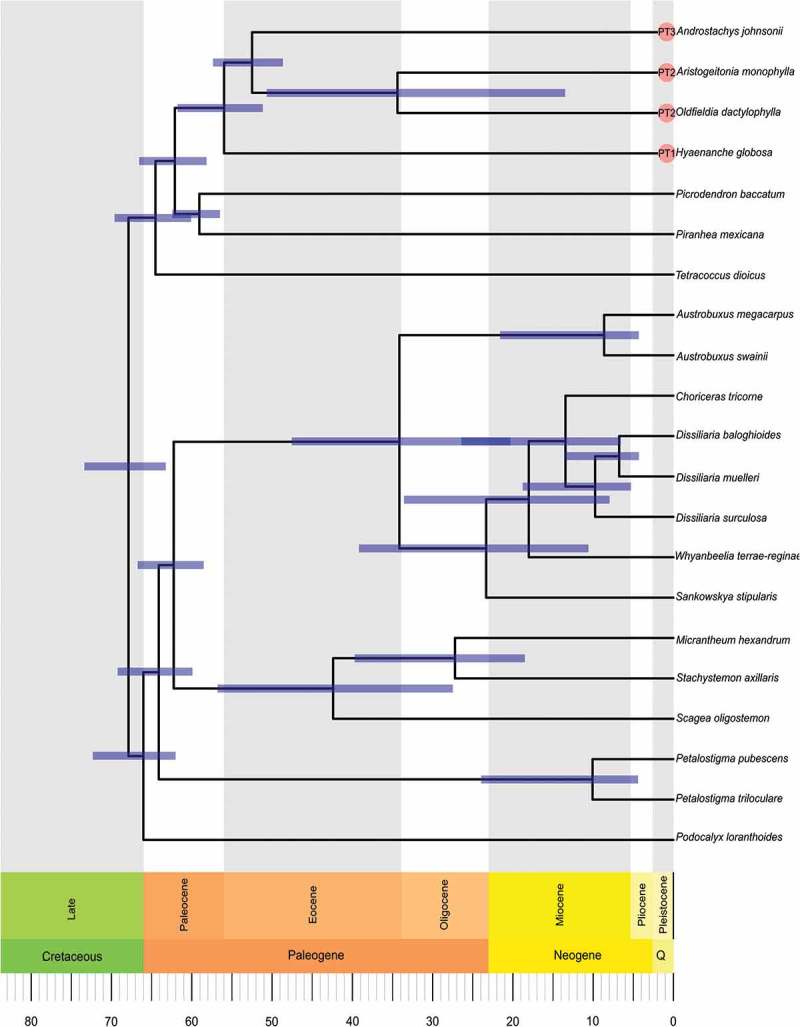
Maximum Clade Credibility consensus timetree of Picrodendraceae obtained using the fossilised birth–death prior. All fossils (see Table VII) have been trimmed off after the construction of the consensus tree. Pollen types for the Afro-Indian taxa are indicated. Bars indicate 95% highest posterier density (HPD) intervals.

The respective ages of the different Picrodendraceae lineages in the dated phylogeny (Figure 25) support a primary dispersal from South America to Australasia via Antarctica. The Australasian clade supposedly originated between the Late Cretaceous and the Paleocene (69.2–59.9 Ma). The Afro-Indian clade apparently split from the *Piranhea-Picrodendron* clade between the latest Cretaceous and the Paleocene (66.8–58.1 Ma; Figure 25). The age of the Afro-Indian crown group spans between the Paleocene and the Eocene (61.8–51.1 Ma). These relatively young ages are in agreement with the fossil records and suggestive of a boreotropical distribution with dispersal across the North Atlantic Land Bridge. However, since the earliest fossil records representing the Afro-Indian clade are mostly from European localities, that could bias the inferred splits towards younger ages by missing potential diversity in Afro-India. Similar biases have been reported for other groups of southern origin with mostly European records, like Solanaceae (Wilf et al. 2017).

If results are accepted from the analysis of the current African fossil records and the dated phylogeny (Figure 25) indicating PT 2 pollen (occurring in present day *Aristogeitonia, Mischodon, Oldfieldia, Voatamalo*) did not evolve until the early to middle Miocene, then dispersal of that lineage from continental Africa to Madagascar and other islands on the eastern side of Africa, and especially to India must have taken place via island hopping and/or long-distance dispersal. It is speculative, but possible that birds transported seeds from the continent, or that seeds along with ants (their primary disperser) and/or even mammals or reptiles, were transported eastwards on floating debris, by one or more successive dispersal events.

Alternatively, the lack of fossil Picrodendraceae pollen from the Eocene to Oligocene of continental Africa may not be because these plants did not thrive in these parts of the world during that time, but more due to the fact that not enough samples have been studied or that they have simply been overlooked or misidentified due to their superficial similarities to e.g. Asteraceae, Malvaceae and Arecaceae. The fossil pollen record to date (Table VII) has been compiled by the Vienna working group, routinely using SEM, and the Australasian palynological community. Palynologists working in Africa may not yet be fully aware of the presence of this pollen group or of its affiliation. This is supported by the current identification of the Miocene Mush MT pollen in the Mush Valley palynoflora (Ethiopia), where this pollen type was most likely previously referred to Myristicaceae (Danehy 2010), and the Saldanha MT (*Hyaenanche* lineage) pollen in the Saldanha Bay palynoflora (South Africa), a well-studied Miocene flora, where this type of pollen was previously referred to *Baumannipollis* with affiliation to Malvaceae (Roberts et al. 2017).

## Conclusion and outlook

Picrodendraceae produce unique pollen grains that are easily identified using the combination of LM and SEM. The extant Afro-Indian pollen can be grouped into three morphological types (geographical lineages). The PT 1 occurring in *Hyaenanche* is clearly the basal most surviving Afro-Indian pollen type that gave off subsequent pollen types (PT 2 and PT 3).

Combining evidence from the pollen morphology of Picrodendraceae along with their fossil records, the current phylogenetic framework, a new dated intra-familial phylogeny, and relevant paleogeographic scenarios from the Late Cretaceous to early Cenozoic, we conclude that: (1) Picrodendraceae originated in the Americas, (2) the family dispersed from Southern America across Antarctica and into Australasia during the late Paleocene, (3) a second migration (of a different lineage) took place from the Americas across the North Atlantic Land Bridge and into Europe during the Paleocene and/or Eocene, (4) the Picrodendraceae reached their maximum north–south distribution prior to the end of the Eocene, (5) the family dispersed into Africa from Europe during the Eocene, (6) and dispersed from continental Africa to islands east of the continent and to India in the Neogene via island hopping or long distance dispersal.

Still, to fully understand the early divergence and evolution of Afro-Indian Picrodendraceae, palynological studies need to focus on Late Cretaceous to early Cenozoic floras from the Americas and Afro-India using the morphological data presented here. This will enable scientists to correctly affiliate pollen of this group to particular lineages and to fill some of the gaps in the family’s early history. Also, the expansion of the molecular phylogeny is needed to more completely understand the historical evolutionary relationship of all the Afro-Indian, American and Australasian taxa of this family.

## Supplementary Material

Supplemental Material Table S1
